# A robotic system for automated genetic manipulation and analysis of *Caenorhabditis elegans*

**DOI:** 10.1093/pnasnexus/pgad197

**Published:** 2023-07-05

**Authors:** Zihao Li, Anthony D Fouad, Peter D Bowlin, Yuying Fan, Siming He, Meng-Chuan Chang, Angelica Du, Christopher Teng, Alexander Kassouni, Hongfei Ji, David M Raizen, Christopher Fang-Yen

**Affiliations:** Department of Bioengineering, School of Engineering and Applied Science, University of Pennsylvania, Philadelphia, PA 19104, USA; Department of Bioengineering, School of Engineering and Applied Science, University of Pennsylvania, Philadelphia, PA 19104, USA; Department of Bioengineering, School of Engineering and Applied Science, University of Pennsylvania, Philadelphia, PA 19104, USA; Department of Bioengineering, School of Engineering and Applied Science, University of Pennsylvania, Philadelphia, PA 19104, USA; Department of Bioengineering, School of Engineering and Applied Science, University of Pennsylvania, Philadelphia, PA 19104, USA; Department of Bioengineering, School of Engineering and Applied Science, University of Pennsylvania, Philadelphia, PA 19104, USA; Department of Bioengineering, School of Engineering and Applied Science, University of Pennsylvania, Philadelphia, PA 19104, USA; Department of Bioengineering, School of Engineering and Applied Science, University of Pennsylvania, Philadelphia, PA 19104, USA; Department of Bioengineering, School of Engineering and Applied Science, University of Pennsylvania, Philadelphia, PA 19104, USA; Department of Bioengineering, School of Engineering and Applied Science, University of Pennsylvania, Philadelphia, PA 19104, USA; Department of Biomedical Engineering, College of Engineering, The Ohio State University, Columbus, OH 43210, USA; Department of Neurology, Perelman School of Medicine, University of Pennsylvania, Philadelphia, PA 19104, USA; Department of Bioengineering, School of Engineering and Applied Science, University of Pennsylvania, Philadelphia, PA 19104, USA; Department of Neuroscience, Perelman School of Medicine, University of Pennsylvania, Philadelphia, PA 19104, USA; Department of Biomedical Engineering, College of Engineering, The Ohio State University, Columbus, OH 43210, USA

**Keywords:** experimental organisms, model invertebrates, *Caenorhabditis elegans*, genetic techniques, automated methods

## Abstract

The nematode *Caenorhabditis elegans* is one of the most widely studied organisms in biology due to its small size, rapid life cycle, and manipulable genetics. Research with *C. elegans* depends on labor-intensive and time-consuming manual procedures, imposing a major bottleneck for many studies, especially for those involving large numbers of animals. Here, we describe a general-purpose tool, WormPicker, a robotic system capable of performing complex genetic manipulations and other tasks by imaging, phenotyping, and transferring *C. elegans* on standard agar media. Our system uses a motorized stage to move an imaging system and a robotic arm over an array of agar plates. Machine vision tools identify animals and assay developmental stage, morphology, sex, expression of fluorescent reporters, and other phenotypes. Based on the results of these assays, the robotic arm selectively transfers individual animals using an electrically self-sterilized wire loop, with the aid of machine vision and electrical capacitance sensing. Automated *C. elegans* manipulation shows reliability and throughput comparable with standard manual methods. We developed software to enable the system to autonomously carry out complex protocols. To validate the effectiveness and versatility of our methods, we used the system to perform a collection of common *C. elegans* procedures, including genetic crossing, genetic mapping, and genomic integration of a transgene. Our robotic system will accelerate *C. elegans* research and open possibilities for performing genetic and pharmacological screens that would be impractical using manual methods.

**Significance Statement**

The nematode *Caenorhabditis elegans* is a powerful genetic model organism in life sciences due to its compact anatomy, short life cycle, and optical transparency. Current methods for worm genetics rely on laborious, time-consuming, and error-prone manual work. Here, we describe a general-purpose automated tool capable of genetically manipulating *C. elegans*. Our robotic system will accelerate a broad variety of *C. elegans* research and open possibilities for performing genetic and pharmacological screens that would be impractical using manual methods.

## Introduction

Classical genetics, which investigates the heritability of traits across generations, usually requires manipulating reproductive behaviors of organisms and inferring their genetic properties by assaying their traits. The microscopic nematode *Caenorhabditis elegans* is one of the most widely used genetic models in life sciences due to its easy maintenance, optical transparency, and rapid life cycle (1, 2). Studies in *C. elegans* have pioneered major fundamental advances in biology, including those in programmed cell death (3), aging (4), RNA interference (5), and axon guidance (6). Work with worms has pioneered important techniques in modern biology including genome sequencing (7), cell lineage tracing (3), gene editing (8), electron microscopic reconstruction of neural connectivity (9), demonstration of green fluorescent protein (10), and optogenetic manipulation of neural activity (11).

Genetic manipulations in *C. elegans* are performed by manual procedures, which involve identification of animals under a microscope and transfer of worms or embryos from one agar plate to another using a wire pick (12). While manual procedures are reliable and technically simple, they have important limitations.

Manual methods are labor intensive since they require animals to be manipulated individually. This presents challenges to experiments that require thousands of groups to be managed, for example conducting genetic screens (13), working with collections of wild isolates (14), or dealing with mutagenized strains of the Million Mutation Project (15).

During design of lab experiments, the use of manual procedures creates practical limits to the number of conditions and the number of replicates for each condition, weakening statistical power. Standard population sizes used for *C. elegans* lifespan experiments have been shown to be underpowered for moderate differences in lifespan between groups (16).

Finally, manual approaches require training and are prone to errors. This reliance on investigator-learned skills imposes a barrier to entry for scientists without *C. elegans* experience wishing to use this system.

To address the limitations of manual methods, automated organism manipulations have been reported for bacteria and single cells in the field of synthetic biology (17) and for adult *Drosophila* (18). For work with *C. elegans*, automated imaging systems have been developed for behavioral analysis (19), lifespan or healthspan measurement (20–22), and drug screening (23). Microfluidic (24–28) and flow-cell devices (29, 30) have been demonstrated for high-throughput animal imaging and sorting. However, to our knowledge, no methods for automated genetic manipulations in *C. elegans* have been reported.

Here, we present WormPicker, a general-purpose robotic system allowing automated phenotyping and genetic manipulation of *C. elegans* on agar substrates, using techniques resembling manual methods. Our device contains a 3D motorized stage carrying a robotic arm and an optical system. The robotic arm manipulates animals using a thin, electrically sterilized platinum wire loop. Analogous to manual methods, the robotic arm picks worms by performing spatially and temporally controlled motions above and on the agar surface, using food bacteria to encourage the worm to adhere to the loop. Contact between the platinum wire loop and the agar surface is perceived by a capacitive touch sensing circuit, providing feedback in conjunction with the imaging system for fine adjustment of the pick trajectory relative to the animal.

The robot's optical system is capable of monitoring animals over an entire plate at low magnification [6-cm diameter circular field of view (FOV)] while simultaneously imaging individual animals at high magnification (1.88 mm × 1.57 mm FOV) to obtain more detailed morphological and/or fluorescence information. Using machine vision methods, worms can be recognized and tracked over the plates in low magnification and undergo detailed phenotyping in high magnification across different attributes, including developmental stage, morphology, sex, and fluorescence expression. We developed system control software through which the user can specify multistep genetic procedures to be performed.

Using these automated tools, we successfully carried out three genetic procedures commonly performed in *C. elegans* research. First, we generated a genetic cross between transgenic and mutant animals using a classic genetic hybridization scheme. Second, we performed genetic mapping of a genome-integrated fluorescent transgene. Finally, we integrated an extrachromosomal transgenic array to the genome, creating stable transgenic lines. Successful completion of these complex genetic procedures demonstrates WormPicker's effectiveness and versatility as a broadly useful tool for *C. elegans* genetics.

## Results

### Overview of WormPicker system design

**Fig. 1.**
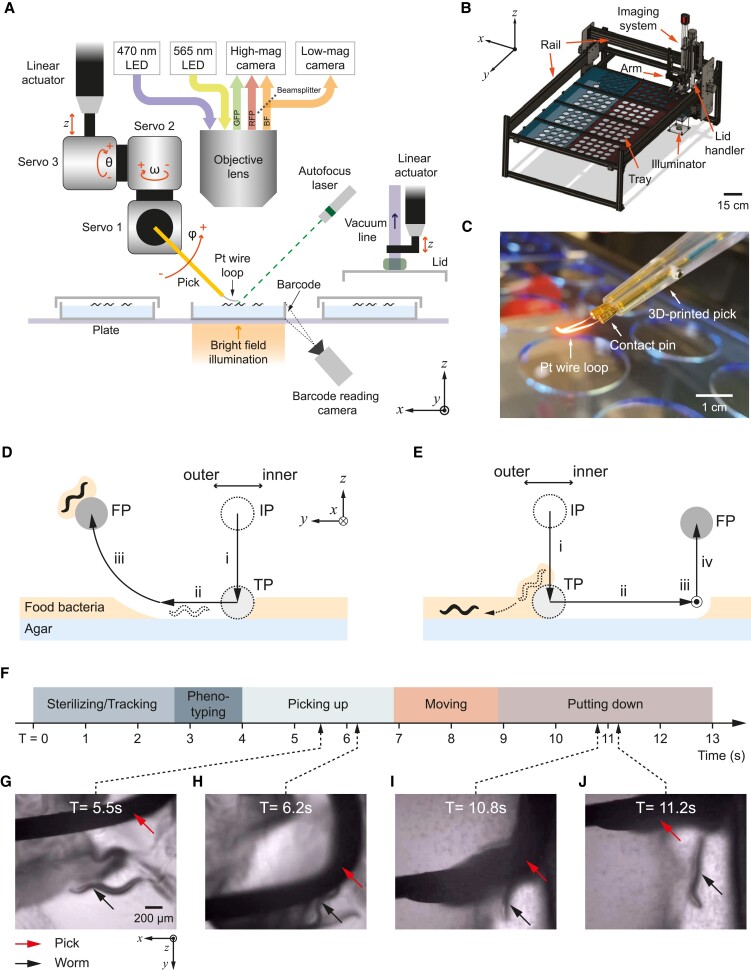
WormPicker system configuration. A) WormPicker geometry. *C. elegans* is manipulated by a wire pick motorized by servo motors and a linear actuator. Animals are imaged by an optical imaging stream. Lids are manipulated by a vacuum grabbing system. The imaging and manipulation assemblies are mounted on a 3D motorized stage. B) WormPicker system overview. C) Photograph of the 3D-printed pick, captured when the wire loop is being sterilized by an electric current. D and E) Schematic of pick motion trajectories for *C. elegans* picked up from and put down to agar substrate. IP: initial position; TP: agar-touching position; FP: final position. F) Timeline for stages throughout the automated transfer. G–J) Key frames captured by the high-magnification camera during the Picking up (G and H) and Putting down stage (I and J).

Our system contains a robotic picking arm, optical imaging system, lid manipulators, and other elements mounted on a 3D motorized stage to work with an array of up to 144 agar plates (Figs. 1A and B and S1).

The array of plates is housed on a platform (Fig. 1B), containing eight trays that can be prepared separately and then slid into the automated system (Fig. S2A and B). Each plate on the tray can be labeled with an adhesive barcode and text (Figs. 1A and S2C and D). We developed software to track plates during experiments using their barcode identifiers (Fig. S2G–I).

The imaging system is designed for bright field and fluorescence imaging through low- (0.035×) and high-magnification (10×) optical pathways. The low-magnification stream has a 6-cm diameter circular FOV, capable of imaging one 6-cm diameter plate; the high-magnification stream has a 1.88 mm × 1.57 mm FOV, capable of imaging individual animals in detail. For bright field imaging, light is collected by an objective lens and divided into low- and high-magnification imaging streams by a beamsplitter (Figs. 1A and S3A). For fluorescence imaging, collimated excitation LEDs (center wavelengths 470 and 565 nm) and fluorescence optics (Fig. S3B and C) enable imaging in green (503- to 541-nm wavelength) and red (606- to 661-nm wavelength) channels (Movie S1) via the high-magnification pathway.

To quickly bring the surface of a plate into focus, we use a laser-based autofocusing system (Figs. 1A and S3D). A 532-nm laser pointer with an integrated cylindrical lens generates a line projected onto the agar surface from an oblique angle, providing feedback for the motorized stage to move the imaging system to the correct position.

Plate lids are manipulated by a vacuum gripping system (Figs. 1A and S3E). Two lid handlers are mounted to either side of the main motorized gantry for manipulating lids for a source plate and a destination plate separately (only one visible in Fig. 1A). Each vacuum lid gripper is raised and lowered by a motorized linear actuator (Movie S2).

The robotic picking arm (Figs. 1A and S3F) contains a motorized linear actuator for fine adjustment of the pick's height and three servo motors to provide rotational degrees of freedom. The 3D-printed pick contains a platinum wire loop attached to its end (Fig. 1C), for manipulating *C. elegans* in a manner analogous to manual methods (12).

Conventional pick sterilization prior to manipulating *C. elegans* on agar substrates requires an open flame, which poses safety risks in an automated system. We adopted an electric sterilization approach (Fig. 1C) in which a current is passed through the wire loop to sterilize it via resistive heating (Movie S3).

Picking up *C. elegans* requires very fine control of the pick to avoid damage to the worm or the agar surface. While the horizontal position of the pick can be monitored by the imaging system, its height relative to the agar surface is more difficult to determine. To address this problem, we developed a capacitive touch sensing circuit (Fig. S3G) that detects contact between the platinum wire and agar surface and provides feedback for fine tuning the pick's movements. Additionally, the pick's height relative to the optimal focus of the imaging system can be monitored by measuring the intensity of the object (Fig. S4).

To pick up a worm (Fig. 1D), the wire pick is positioned above the agar [initial position (IP)], with a horizontal offset to the target worm. The linear actuator lowers the pick until contacting the agar surface [touching position (TP)] as perceived by the capacitive touch sensor (phase i). Next, Servo 2 horizontally swipes the pick on the agar surface (phase ii). Servos 1 and 3 act simultaneously to perform a curved motion (phase iii) for picking up the target animal using the outer side of the wire loop. To release the worm from the pick to the substrate (Fig. 1E), the pick is positioned above the agar (IP); the linear actuator vertically lowers (phase i) the pick until it touches the agar (TP); the Servo 2 horizontally swipes (phase ii) the pick on the agar surface, to release the worm from the pick. The basic pick-up and put-down actions are chained in series for automated *C. elegans* transfer (Fig. 1F). Figure 1G–J and Movie S4 depict these operations as observed by the WormPicker imaging system; Movie S5 depicts these actions as observed by an external camera.

### Machine vision enables automated identification and phenotyping

**Fig. 2.**
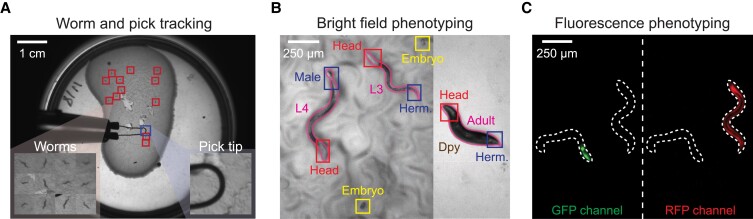
Overview of machine vision approaches. Machine vision algorithms were developed for processing images acquired from the (A) low- and (B and C) high-magnification imaging streams. The low-magnification frames (A) are analyzed for tracking worms and the pick in real time. The high-magnification bright field images (B) are processed by a set of Mask-RCNNs for close inspection of individual animals, including contour segmentations and assays of developmental stage (including embryo), sex, and morphology. The high-magnification fluorescence images (C) are subject to intensity analysis for identifying fluorescent markers in GFP and RFP channels. Dashed line: animal contours segmented in the bright field.

We developed machine vision analysis software for the low- and high-magnification imaging streams. We analyze the low-magnification images (Fig. 2A) using a combination of convolutional neural networks (CNNs) and motion detection for tracking animals and the pick in real time. High-magnification bright field images (Fig. 2B) are analyzed by a set of Mask-Regional CNNs (Mask-RCNNs) (31) capable of performing pixel-wise object segmentations. Animals’ contour geometries are analyzed for developmental stage and morphology. In addition, we trained separate networks for sex determination and embryo detection. Inferences from multiple Mask-RCNNs are integrated for assaying phenotypes for individual animals over different attributes.

For high-magnification fluorescence images (Fig. 2C), we perform intensity analysis to extract the valid fluorescent signals from the background. To assay fluorescence expression, the fluorescence images are correlated with the animal contours segmented using the bright field images (Fig. 2C).

Details of our machine vision approaches are given in Supplementary materials, Extended Methods and Fig. S5.

### WormPicker reliably picks *C. elegans* of various stages and phenotypes

First, we asked whether the automated picking causes damage to *C. elegans*. We used our system to pick animals of all stages, ranging from L1 larvae to day 5 adults, and an array of mutants, including *lin-15* (multivulva), *rol-6* (roller), *unc-13* (uncoordinated and paralyzed), and *lpr-1* (fragile cuticles) (32). We measured the number alive 24 h after the automated picking. As a control, we repeated the procedure using the standard manual methods (12). We observed that animals manipulated by WormPicker showed viability comparable with that of standard methods (Table S1).

We asked how effectively the system picks up and puts down animals. We manually verified the success of individual pick-up and put-down attempts through the live image stream. We observed success rates ∼90% for picking up and putting down different types of animals (Table S2). When working with unseeded plates, we precoated the wire loop with bacteria and observed similar success rates as for seeded plates.

These experiments show that WormPicker is safe and effective for transferring many different types of *C. elegans*, including young, aged animals, and various mutants.

### Automated throughput is comparable with that of experienced researchers

To compare the rate of automated and manual picking, we evaluated how quickly the robot and human researchers could perform a fluorescent animal sorting task. We used a strain in which some but not all worms carry a *myo-2::GFP*-labeled fluorescent extrachromosomal array (YX256); the task was to sort these worms into two plates containing fluorescent and nonfluorescent animals.

The robotic system sorted the animals (of mixed stages) with a throughput of 3.21 ± 0.66 (mean ± SD) animals per minute (APM).

We recruited a group of *C. elegans* researchers (*N* = 21) to perform the same task using standard methods (12). The mean and median years of their *C. elegans* experience were 7.61 and 5 years, respectively. We tasked each volunteer to sort ∼20 animals (of mixed stages) under a fluorescence stereoscope. Both WormPicker and the researchers picked individual worms and sterilized the pick between transfers. The measured throughputs are plotted versus years of experience in Fig. S6. The manual picking throughput was 3.56 ± 1.67 (mean ± SD) APM.

These results show that the throughput of the robotic system for this fluorescent animal sorting task is comparable with that of experienced human researchers.

### The automated system maintains an aseptic environment

As in manual work, it is important to minimize contamination of media in our automated system. We designed the WormPicker to maintain an aseptic environment. We built the system inside a panel enclosure to prevent airborne contaminants from entering. We sanitized the active components, including the robotic arm, microscope, and plate trays using 70% ethanol before experiments. During experiments, plates had lids on for most of the time, except briefly during picking operations. For the experiments reported in Tables S1 and S2, we did not observe any plate contaminated 10 days after being manipulated by WormPicker (*N* = 101 plates). In comparison, 3.4% of the plates were contaminated after manual picking in the same room (*N* = 119 plates). These results show that our protocols were sufficient for maintaining an aseptic environment for the experiments.

### Scripting toolsets enable complex genetic manipulations

In order for WormPicker to be useful for practical laboratory work, the basic elements of identifying and transferring worms need to be combined to form complex genetics procedures. To that end, we developed system control software, WormPickerControl (Fig. S7A). An application programming interface (API) enables the user to specify *C. elegans* procedures to be carried out by the automated system. We developed a library of source scripts, each responsible for a specific task ranging from simple to complex. The automated system catalogs a set of plates based on their barcode identifiers (Figs. 1A and S2) and stores their information in a database.

**Fig. 3.**
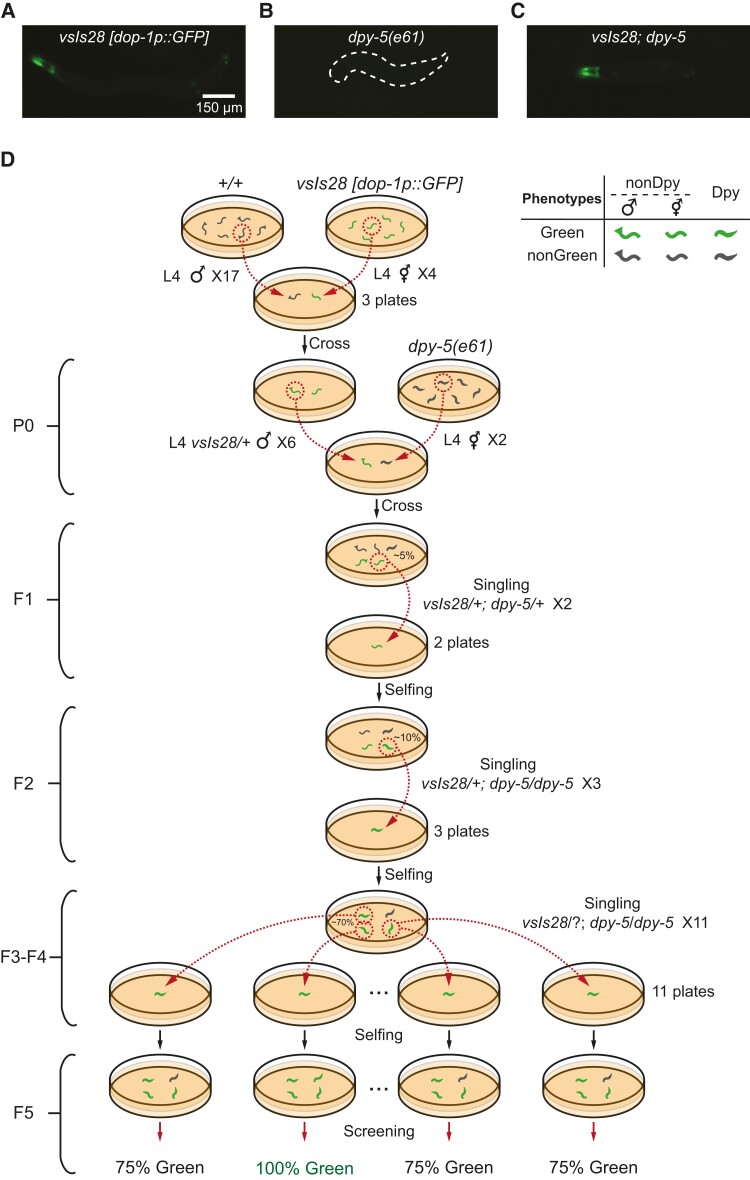
Automated genetic cross in *C. elegans*. WormPicker generated a genetic cross between animals carrying an integrated green fluorescent transgene *vsIs28 [dop-1p::GFP]* and *dpy-5(e61)* mutants. A–C) GFP expression in the strain carrying (A) *vsIs28*, (B) *dpy-5*, and their hybridization (C) *vsIs28*; *dpy-5*. D) Schematic of the genetic cross.

**Fig. 4.**
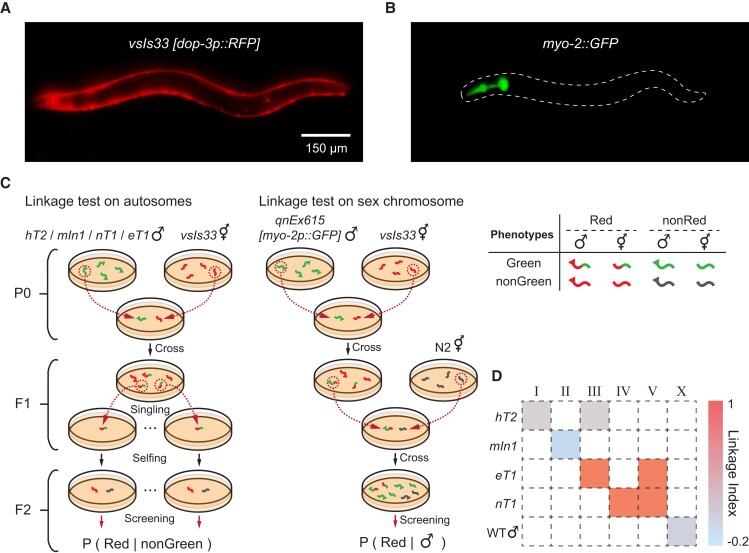
Automated genetic mapping of a red fluorescent transgene in *C. elegans*. A) RFP expression in the strain carrying the transgene of interest *vsIs33 [dop-3p::RFP]*. B) GFP expression in the genetic balancer strains carrying *myo-2::GFP* markers. C) Schematic of the linkage test. D) Linkage of *vsIs33* over chromosomes, quantified by Linkage Indices shown in a heat map. *x* axis: indices of the chromosomes; *y* axis: list of the genetic balancers.

**Fig. 5.**
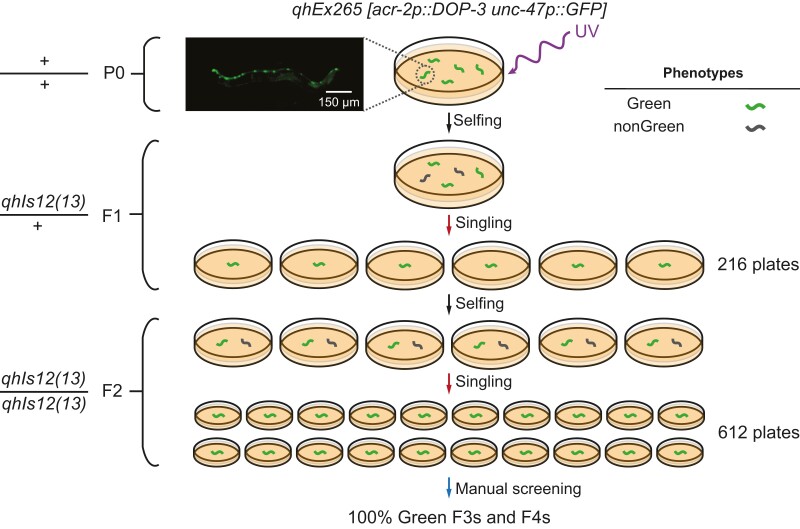
Automated genomic integration of a green fluorescence-labeled extrachromosomal array *qhEx265 [acr-2p::DOP-3 unc-47p::GFP]*. The image shows the GFP expression of the array. *qhIs12* and *qhIs13*: names of the potential integrated transgenic alleles.

Using WormPickerControl, we developed and tested a collection of genetic procedures commonly undertaken in *C. elegans* labs (Figs. 3–5). These are not intended to be a complete set of genetic protocols but rather to provide a useful framework that can be modified and adapted to other experiments as needed.

### Automated genetic cross

The genetic cross, by which two or more mutations or transgenes are combined, is performed by placing males together with hermaphrodites on the same agar plate (33). While monitoring the plates, researchers pick cross-progeny with desired phenotypes. Most genetic crosses require manipulating animals over multiple generations.

We automated a genetic cross between two *C. elegans* strains: *dop-1p::GFP* fluorescent transgenic (LX831) and a *dpy-5(e61)* mutant (CB61) (Fig. 3). The LX831 strain contains an integrated transgene *vsIs28 [dop-1p::GFP]* and has a Green phenotype; i.e. GFP is expressed in several cell types including some neurons and muscles (Fig. 3A). The *dpy-5* mutant has a Dumpy (Dpy) phenotype, characterized by a morphology that is shorter and stouter than wild-type animals (Fig. 3B). The *vsIs28* transgene is dominant (i.e. both heterozygotes and homozygotes show green fluorescence) whereas the *dpy-5* mutation is recessive (i.e. only the homozygotes show the Dpy phenotype). The resulting hybridized strain displays both phenotypes of two parental strains; i.e. it is both Green and Dpy (Fig. 3C).

Figure 3D depicts a schematic of the genetic cross. WormPicker picked 17 L4 males from a wild-type (N2) population that consisted of a mixture of males and hermaphrodites. It also picked four L4 hermaphrodites from the LX831 strain to the same plates. Green males were visible in the progeny, indicating that the mating was successful. Next, six Green L4 *vsIs28* heterozygous males and two *dpy-5* homozygous L4 hermaphrodites were picked onto a plate for mating. We consider this mating the parental (P0) generation.

Filial generation 1 (F1) progeny included animals that were Green and not Dpy. These were the desired worms heterozygous for both *vsIs28* and *dpy-5* and were therefore transferred by WormPicker to fresh plates. The automated system screened over the plate and singled two F1 hermaphrodites with the wanted phenotypes.

Four phenotypes were observed in the F2 generation, including Dpy-Green, Dpy-nonGreen, nonDpy-Green, and nonDpy-nonGreen. The frequency of the desired Dpy-Green phenotype was ∼10%. WormPicker inspected the F2 populations and singled three Dpy-Green animals.

F3 populations descending from each of the three F2s were 100% Dpy but only 75% Green indicating that they were homozygous for *dpy-5* but heterozygous for *vsIs28*. WormPicker then singled 11 Dpy-Green animals.

The automated system screened for the percentage of Green F5s descending from each of the eleven F4s and identified one line displaying homozygous fluorescent; i.e. 100% of F5s were Green.

The results were verified by an experienced *C. elegans* researcher by noting that the resulting strain was positive for both Green and Dpy phenotypes and that these phenotypes bred true in subsequent generations.

### Automated genetic mapping of a transgene

The identification of the genotype causing a particular phenotype usually requires genetic linkage analysis. The first step in such analysis is to identify the chromosomes harboring the genetic change.

*C. elegans* has six chromosomes, of which five are autosomes and one is the X chromosome. Hermaphrodites are diploid for all six chromosomes, while males are diploid for five autosomes and haploid for the X chromosome.

Genetic mapping in *C. elegans* can be performed by setting up genetic crosses between the strain of interest and a set of marker strains and measuring the segregation pattern between the marker phenotypes and the phenotype of interest.

We used WormPicker to perform an automated genetic mapping of an integrated red fluorescent transgene *vsIs33 [dop-3p::RFP]* (LX811) (Fig. 4). This strain has a Red phenotype; i.e. RFP is expressed in cells expressing DOP-3 dopamine receptors (Fig. 4A).

We evaluated the linkage of *vsIs33* to autosomes by setting up crosses between our strain of interest (LX811) and a set of balancer strains. These balancer strains are labeled by *myo-2::GFP* markers and have a Green phenotype (GFP expressed in pharyngeal muscles) (Fig. 4B). We used the reciprocal translocations *hT2* (34) *[qIs48] (I;III)*, *eT1* (35, 36) *[umnIs12] (III;V)*, and *nT1* (37–39) *[qIs51] (IV;V)* to test for linkage to large portions of chromosomes I and III, III and V, and IV and V, respectively; we also used the inversion *mIn1* (40) *[mIs14] (II)*, to test for linkage to chromosome II (Fig. 4C and Supplementary material, Extended Methods and Figs. S8–S11).

WormPicker picked L4 Green males from the balancer strains and hermaphrodites from the strain of interest (LX811) for mating. WormPicker then screened for F1 Red-Green hermaphrodites which were subsequently singled. The double-fluorescent F1 hermaphrodites self-fertilized and produced F2s, where the percentages of Red among nonGreen animals were assessed to test for linkage between *vsIs33* and particular autosomes. According to the classic genetic theory, linkage of the transgene to the tested balancer chromosomes would yield 100% of nonGreen animals to be Red, whereas nonlinkage of the transgene to the balancer would be reflected by 75% Red among the nonGreen progeny.

For testing linkage to the X chromosome, WormPicker generated a genetic cross between LX811 hermaphrodites and wild-type males harboring a dominant extrachromosomal transgene *qnEx615 [myo-2p::GFP]* (NQ1155) (Supplementary material, Extended Methods), which helped us to identify F1 cross-progeny. As shown in Fig. 4C, F1 Red-Green males were picked out for mating with N2 (wild-type) hermaphrodites. F2s were screened by the robotic system, and the observed percentage of Red among males was used for quantifying the degree to which *vsIs33* is linked to the X chromosome. The theory predicts that none of the F2 males would be Red if X-linked and that 50% of the F2 males would be Red if not X-linked (Fig. S12).

To quantify the strength of linkage, we defined a Linkage Index ranging from −1 to 1, where 1 implies the strongest possible linkage and 0 the weakest (Supplementary material, Extended Methods). Our data (Fig. 4D) indicate a strong linkage of *vsIs33* both to *eT1* and *nT1*, and no linkage to *hT2*, to *mIn1*, or to the X chromosome. Since both *eT1* and *nT1* balance a large portion of chromosome V, our results imply that the RFP transgene is located on chromosome V.

### Genomic integration of a transgene

Transgenic *C. elegans* are usually generated by microinjection, through which cloned DNAs are delivered to the distal gonadal arm, forming extrachromosomal transgenic arrays (41, 42). To provide stable inheritance and expression, the transgenic arrays can be integrated to the genome. A proven method for genomic integration is to irradiate the strain of interest, causing breakage in chromosomes, which triggers DNA repair, a process through which the transgenic arrays can be ligated to the chromosomes by chance (41). Due to its low frequency, identifying animals with the transgene integrated requires isolating at least several hundred individual worms and screening for 100% inheritance of the transgene in subsequent generations, a highly labor-intensive task (43). In particular, the need to single hundreds of animals can consume a substantial amount of time, even for experienced *C. elegans* researchers.

Using WormPicker, we performed a genomic integration of an *acr-2p::DOP-3* extrachromosomal array labeled by a green fluorescent marker *unc-47p::GFP* (YX293) (Fig. 5). The strain displayed ∼65% transmission of the transgenic array to the next generation. To create the P0 worms, we irradiated 68 L4s with ultraviolet light (254 nm, 30 mJ/cm^2^). WormPicker isolated 216 F1s to individual plates. From these plates, the automated system isolated 612 of their progenies (F2s) to individual plates. The F2 animals were manually screened for 100% transmission of the transgene to F3s and F4s. Among these, two lines were identified as potential independent integrants. For both lines, 100% transmission of the transgene was confirmed over several subsequent generations, consistent with successful array integration.

## Discussion

In this work, we have demonstrated that WormPicker can automate a variety of *C. elegans* genetic procedures usually performed by manual methods. In addition, our scripting tools provide flexibility for carrying out customized experiments as well as integrating the system into diverse genetic screens and analyses, potentially including pharmacological screening (44, 45), screening for aging phenotypes (4, 46, 47), and studies of natural genetic variation (48).

Robotic manipulation of *C. elegans* opens possibilities for experiments that would be difficult or impractical for manual methods, especially for those involving a large number of strains or conditions. For example, our laboratory is using this machine to perform a genetic screen for modifiers of stress-induced sleep (49).

The deep-learning-aided machine vision methods we developed here, capable of segmenting individual animals and assaying them across different attributes, may find other applications for *C. elegans* studies, for example, in analyses of locomotion (50, 51), aging (52, 53), and sleep (54).

Our self-sterilizing loop design can be used for automatically manipulating other microscopic organisms, for example, other nematodes, *Drosophila* larvae, bacteria, and fungi.

Our WormPicker system is primarily made from off-the-shelf components. We have provided a component list including estimated material costs (Table S3) and design files for the custom-made parts (Design File S1). The system control software is accessible at an online repository (55).

It is important to acknowledge the limitations of our work. For a simple fluorescent animal sorting task, the throughput achieved by WormPicker with a single robotic arm was somewhat smaller than the average of experienced researchers. However, it has the advantage of being able to work continuously without fatigue and to conduct a large number of operations in parallel.

Animals sometimes cluster together in a way that makes it difficult to pick a single individual. We have demonstrated that WormPicker's machine vision is able to segment individual animals on highly populated plates (Fig. S6D). For the experiments presented here, we programmed the robot to perform intermediate picking (Supplementary material, Extended Methods) before transferring animals to their destinations, as a method of handling clusters of worms. In addition, brief blue light illumination (52, 56) or plate vibration could be used to disperse clusters of animals.

Although WormPicker's machine vision system works well for recognizing and tracking the wild-type animals and mutants tested here, some modifications of our algorithms may be necessary for some mutants with unusual morphologies and behaviors.

We expect all animal manipulations will continue to involve some degree of manual work in conjunction with the automated system. The researcher must prepare plates, load and unload them on the trays, and select the appropriate operations. The effective integration of WormPicker to lab work will require determining which genetic operations are most amenable to automation and which are better left to manual work. The goal of our work is not to fully automate experiments, in a sense that the human is no longer needed, but rather to provide tools to increase the productivity of the researchers.

## Materials and methods

### Mechanical setup

WormPicker is based on a 1.5 m × 1 m rectangular framing system constructed from aluminum extrusions (OpenBuilds V-Slot 20 mm × 20 mm, 20 mm × 40 mm, and C-Beam 40 mm × 80 mm).

WormPicker's imaging system and picking arm are moved by a motorized stage along three axes: *X* (80 cm travel), *Y* (125 cm), and *Z* (30 cm). In addition, there is a linear carriage under the plate tray that moves the illumination and plate tracking system. All axes are driven by stepper motors (NEMA 23, 1.8° per step). For the *X* and *Y* axes, motors mounted to the carriage drive motion via a belt attached to both ends of the rail. For the *Z* axis, a stepper motor drives motion through the rotation of a lead screw.

The maximum speed of the *X* axis was set to 146.67 mm/s, *Y* axis 146.67 mm/s, and *Z* axis 8.33 mm/s. The maximum acceleration of the *X* axis was set to 120 mm/s^2^, the *Y* axis 120 mm/s^2^, and the *Z* axis 10 mm/s^2^. Stepper motors are controlled through a PC via an OpenBuilds BlackBox motion control system using GRBL firmware.

All aspects of the robotic system were controlled by an Origin PC with an Intel(R) Core i9-10900K CPU at 3.7 GHz and 64 GB of RAM, running Windows 10.

An overview of WormPicker hardware architecture is given in Fig. S1.

### Dual-magnification multimodal optical imaging system

To implement bright field imaging, we constructed an illumination system under the platform, in which light from a white LED is diffused by a ground glass and approximately collimated by a Fresnel lens (Fig. S3A). The low- and high-magnification imaging paths share a common objective lens (achromatic doublet, focal length 100 mm). Light is collected by the objective lens and then divided into the low- and the high-magnification imaging streams by a beamsplitter (ratio of transmission:reflection is 90%:10%). The low-magnification image is relayed to a machine vision camera by a camera lens, while the high-magnification image is formed at a CMOS camera through a set of teleconverter lenses and a tube lens. The high-magnification pathway is an infinity-corrected microscopy system that can support both bright field and fluorescence imaging.

For fluorescence imaging (Fig. S3B and C), two collimated excitation LEDs (center wavelengths 470 and 565 nm) and a set of dual-band fluorescence optics (Chroma 59022) were built in the infinity space in the high-magnification pathway. The spectral characteristics of the fluorescence optics were selected to enable both GFP and RFP imaging. The parameters for fluorescence imaging and processing used in our experiments are provided in Table S4.

Switching between the imaging modes can be achieved by digital relay circuits controlling the excitation LEDs and the under-platform illumination (Fig. S3A–C and Movie S1).

### Robotic picking arm

We built a robotic arm (Fig. S3F) for manipulating *C. elegans* on agar media using a wire loop. The robotic picking assembly consists of a linear actuator, three servo motors, and a 3D-printed worm pick (Fig. 1C). The linear actuator fine tunes the *z* height of the picking arm through a linear carriage. The servo motors are chained orthogonally to provide 3 degrees of freedom in rotation (*θ*, *ω*, and *φ*) (Fig. 1A) for the worm pick. The 3D-printed worm pick is mounted to Servo 1. The design allows two copper wires to fit into the pick stem from the proximal end, and these two wires are connected by a portion of looped platinum wire (90% Pt, 10% Ir, 254 *µ*m diameter) at the distal end of the pick (Fig. 1C). The platinum wire is crimped to copper contact pins for attaching to the end of the pick.

To sterilize the pick, the platinum wire loop is connected to a 5-V DC power supply (Fig. S3G). The resulting 4.5-A DC current sent through the wire heats the loop to a temperature we estimate to exceed 1,000°C based on its color (Fig. 1C and Movie S3).

We used the wire loop as a capacitive sensing probe (Fig. S3G) to monitor contact between the wire and the agar surface and to provide feedback for picking trajectories. For the sensing circuit to function properly, the platinum wire loop is first disconnected from the heating circuit. The capacitance change due to the contact is sensed by a capacitive touch sensor (SparkFun, AT42QT1011), the voltage output of which is monitored by a data acquisition device (LabJack).

### Pick motion trajectories for manipulating *C. elegans*

To pick up an animal (Fig. 1D), the wire pick is positioned above the agar, with a *y*-offset to the target worm (IP). The linear actuator vertically lowers (phase i, change in *z*) the pick until contacting the agar surface (TP) as perceived by the capacitive touch sensor. Next, Servo 2 horizontally swipes (phase ii, change in *ω*) the pick on the agar surface. Next, Servos 1 and 3 act simultaneously to perform a curved motion (phase iii, change in *θ* and *φ*) for picking up the target animal using the outer side of the wire loop [final position (FP)]. During phases i–iii, the motor speeds are maximized.

To put down the animal (Fig. 1E), the pick is positioned above the agar surface (IP). The linear actuator lowers (phase i, change in *z*) the pick until touching (TP), monitored by the capacitive touch sensor. Next, Servo 2 horizontally swipes (phase ii, change in *ω*) the pick on the agar surface, during which the worm detaches from the wire; for some cases, the animal stick to the inner side of the wire loop, the moving stage swipes (phase iii) the pick to −x. Finally, Servo 1 raises (phase iv, change in *φ*) the pick from the agar (FP). The motor speeds are tuned down in different phases, and waiting times were added to the phase transitions.

Representative trajectory parameters are listed in Table S5, with the sign convention depicted in Fig. 1A. These parameters can be adjusted for different types of animals and scenarios.

### Measurement of viability after automated and manual picking

We manually measured animals’ viability 24 h after robotic and manual picking. We classified the animals into three categories: alive, dead, and escaped. An animal was classified as alive if it moved actively; dead if it lost mobility; and escaped if we could not find it on the plate. For the paralyzed mutant *unc-13* (CB1091), we determined its viability by observing pharyngeal pumping.

### Measurement of success rates for the pick-up and put-down procedures

To measure how effectively the system picks up and puts down animals, we manually verified the success of individual pick-up and put-down attempts through the live low-magnification image stream. For accurate manual verifications, we limited the number of animals to <30 per plate to reduce the probability of picking from and putting down to crowded areas. A pick-up attempt was deemed successful if the animal disappeared from the FOV; failed, if the animal remained in the FOV; and vice versa for verifying a put-down attempt. The success rates of the pick-up and put-down attempts were calculated by the number of successes divided by the number of attempts made.

### WormPickerControl

For WormPicker to perform useful work, the basic elements of identifying and transferring animals need to be combined to form multistep genetic procedures. For this purpose, we developed system control software, WormPickerControl (Fig. S7A). This software is written in Python for the frontend and C++ for the backend.

The frontend is an API through which the user writes scripts to specify tasks to be performed by WormPicker. We developed utilities in the API for initiating resources, generating scripts, managing a set of scripts, and sending the commands to the backend.

The backend contains a library of source scripts, WormPickerLib, each controlling the hardware to carry out specific tasks. The user can access WormPickerLib through the API and has the flexibility to combine a set of scripts for generating custom protocols. To further improve the speed, we set up multiple threads in the backend for separately handling image acquisition, image processing, hardware control, and script execution.

WormPickerControl contains a database, storing information in CSV format, through which the automated system catalogs a set of plates.

We constructed a Mask-RCNN segmentation server, written in Python, responsible for segmenting images acquired from different cameras. The server imports Mask-RCNN models from multiple locally saved PTH files. We built client–server sockets connecting WormPickerLib to the segmentation server, through which images acquired by the hardware are sent to the corresponding Mask-RCNN models, and the inference results are sent back to the software for subsequent processing.

### WormPickerLib

WormPickerLib is a library containing source scripts for performing various procedures, ranging from simple to complex. According to their complexity, elements in the library are categorized into low-, mid-, and high-level scripts. Table S6 contains brief descriptions for each script in the library.

The low-level scripts enable the system to execute basic actions, such as sterilizing the pick, finding a worm with some desired phenotypes, picking, and putting down the animal. The mid-level scripts are composed of multiple low-level scripts chained in series, e.g. scripts to pick multiple animals with some desired phenotypes from a source to a destination. The high-level scripts are one iteration above the mid-level scripts, enabling the system to carry out complete *C. elegans* genetic procedures. The high-level scripts consist of a group of mid-level scripts arranged in a timed and conditional manner. The user has the flexibility to develop custom procedures using the elements in WormPickerLib.

Taking the genetic mapping experiment (Fig. 4C) as an example, Fig. S7B depicts the representative scripts, along with their key input arguments, used for generating a genetic cross between two strains JK2810 and LX811. We wrote high-level scripts (CrossWorms, SingleWorms, and ScreenPlates) for instructing WormPicker to set up the mating for P0, single F1s, and screen F2s. The execution of CrossWorms relied on calling the mid-level script PickNWorms twice, each time for picking a certain number of males or hermaphrodites to a plate. Similarly, PickNWorms was executed in a sequential manner by iterating through a set of low-level scripts, which combined the basic actions of imaging and manipulation.

### Strains

We cultivated all the *C. elegans* strains used in this study at 20°C on nematode growth medium plates with OP50 bacteria using the standard methods (57). The strains used are described in Table S7. All the experiments were carried out at room temperature.

## Supplementary Material

pgad197_Supplementary_Data

## Data Availability

Source data for this paper are available in Data Set S1. Details of WormPicker key components can be found in Table S3. Design files for WormPicker hardware are available in Design File S1. Source code for WormPickerControl, network files for the machine learning algorithms, example scripts for the procedures described in this work, and data processing scripts for the genetic mapping experiment (Fig. S13) can be found on GitHub (55).
