# Supplementary material for: A robotic system for automated genetic manipulation and analysis of Caenorhabditis elegans
Source: PNAS Nexus. 2023 Jul 5;2(7):pgad197. doi: 10.1093/pnasnexus/pgad197 (PMC10321491; doi:10.1093/pnasnexus/pgad197)
Supplement: pgad197_Supplementary_Data [file pgad197_supplementary_data.zip › Supplementary material.pdf]

Supplementary material for

A robotic system for automated genetic manipulation and analysis of *Caenorhabditis elegans*

Zihao Li, Anthony D. Fouad, Peter D. Bowlin, Yuying Fan, Siming He, Meng-Chuan Chang, Angelica Du, Christopher Teng, Alexander Kassouni, Hongfei Ji, David M. Raizen, and Christopher Fang-Yen

Corresponding author: Christopher Fang-Yen

Email: fangyen.1@osu.edu

This PDF file includes:

Extended Methods

Figures S1 to S13

Tables S1 to S7

Legends for Movies S1 to S5

Legend for Data Set S1

Legend for Design File S1

References for the supplementary material

Other supporting materials for this manuscript include the following:

Movies S1 to S5

Data Set S1

Design File S1

Extended Methods

Plate tray platform

We constructed a plate tray platform for housing an array of agar plates. The platform holds eight slide-in trays (Fig. S2*A*), each of which is made of two laser-cut transparent acrylic boards (upper layer 3 mm thick, lower layer 4.5 mm thick) sandwiching a 16.26 μm thick aluminum foil layer (Fig. S2*B*). The function of the foil layer is to increase the capacitance change detected by the capacitive touch sensing circuit (Fig. S3*G*) when the picking wire loop contacts the agar surface. Each tray is held by framing rails, enabling the user to remove or replace the tray by sliding in and out (Fig. S2*A*). Each tray is locked into place by a turn latch (McMaster-Carr 1579N12). Coordinates of individual wells on the platform are calibrated and stored in a configuration file.

Plate tracking system

We developed methods for tracking a set of plates based on their barcode labels (Fig. S2*C*). The label contains a machine-readable barcode and human-readable text and is attached to the side of the plate (Fig. S2*D*).

We designed the barcode label to contain 24 black bars, each representing one bit by its thickness (Fig. S2*C*): a thick bar encodes 1, and a thin one 0. Starting from the left, the first 20 bits encode a plate ID number as a binary integer; the next 3 bits encode the MOD-8 checksum of the ID number, used by the algorithm to verify its decoding result. The final (24th) bar is always thin, encoding a stop bit.

We built a machine vision module for imaging the barcode from beneath the plate tray (Fig. S2*D*). The barcode label is illuminated by LEDs and a camera captures the image (Fig. S2*E* and *F*).

We developed algorithms (Fig. S2*G-I*) for reading the barcode identifier. To recognize the barcode, we trained a Barcode-finding Mask Regional Convolutional Neural Network (1) (BcMRC). We curated a training dataset containing 520 images (648 pixels x 486 pixels size and

5.8 cm x 4.4 cm FOV) in which the contours of individual barcodes were manually labeled. BcMRC was adapted from a pretrained ResNet-50-FPN backbone (1). The training was performed with a Stochastic Gradient Descent (SGD) optimizer and the model converged after 9 epochs. The model generates a contour segmentation for the object most likely to be the barcode in the image (Fig. S2*G*) (we assume there is only one barcode visible in the image). The mean Intersection over Union (IoU) score of the mask prediction with respect to the ground truth is 0.942 (N = 80).

We generate a scanning line running through the BcMRC-predicted mask (Fig. S2*G*). Intensities of some pixels above and below the scanning line are averaged for calculating the intensity profile (Fig. S2*H*). Then, the intensity profile is binarized by a threshold H (Fig. S2*I*), $H = (p_{0.1} + p_{0.9})/2$, where $p_{0.1}$ and $p_{0.9}$ are the 10th and 90th percentile intensity values. After the thresholding, a bit is decoded as 1 if the width of the black bar is larger than its complementary white bar right next to it, and 0 otherwise (Fig. S2*I*). The decoded ID number is verified by its checksum. An error is reported if the verification fails.

Individual plates are cataloged in a database (Fig. S7*A*) based on their ID numbers. Information stored in the database includes but is not necessarily limited to the positions on the tray, strain names, genotypes, phenotypes, and histories. The database is arranged in a human and machine-readable CSV format.

Image focus control

The distance between the main objective lens and the agar surface is monitored by a custom-built laser-based level monitor (Fig. S3*D*). A 532 nm laser containing a cylindrical lens generates a collimated laser line lying in the y axis. The laser line is focused by a lens (focal length 100 mm) and projected onto the agar surface at an oblique angle (Fig. S3*D*). Then, the distance between the objective lens and the agar surface can be monitored by the position of the laser line observed by the imaging system. The 3D motorized stage brings the objective to the

correct position, at which the agar surface lines up with its focal plane, by aligning the observed laser line to a previously calibrated position.

Lid handler

Lids are manipulated by two custom-built vacuum grabbers mounted to the 3D motorized stage (Fig. S3*E*). Each grabber consists of a pair of 3D-printed sockets holding a vacuum tube, motorized by a linear actuator (Actuonix, PQ12-30-12-S). A tube fitting is attached to the end of the vacuum line. The height of the vacuum line is controlled by the linear actuator and the vacuum is switched on or off by a solenoid valve (Granzow, H2U29-00Y).

To remove a lid, the main moving gantry positions the vacuum holder above the lid, the solenoid valve activates the vacuum, the linear actuator lowers the vacuum holder, the lid becomes attached by suction to the holder, and the linear actuator raises the holder and the lid (Movie S2). To restore a lid, this process is reversed.

Pick focus control

To bring the pick tip to the optimal focus in the high-magnification FOV, the system monitors the mean intensity of the top 20% darkest pixels $\overline{I_{20}}$ of the image as a measure of focus (Fig. S4). The system sets the height z_f at which the $\overline{I_{20}}$ is minimized as the focus height of the pick tip. The system records the positions read out from the linear actuator and the three servos, i.e., $(l_f, s_{1f}, s_{2f}, s_{3f})$, when the pick tip is at the focus position (x_f, y_f, z_f).

To pick a worm, the system positions the pick tip right above the remembered focus position, i.e., $(x_f, y_f, z_f + \Delta z)$, where Δz is a predefined small amount of height offset (approximately 2 mm). We set the position $(x_f, y_f, z_f + \Delta z)$ to be the initial position (IP in *Main Manuscript*, Fig. 1*D* and *E*), prior to carefully lowering the pick until touching the agar.

We measured the precision of moving the pick tip to the remembered focus position in the x and y directions. The mean differences between the actual position and the focus position are 290 μm (SD = 161 μm) in x and 250 μm (SD = 173 μm) in y (N = 342). This precision allows the

system to move the pick to the focus position directly in a feed-forward manner, helping to improve the worm picking speed.

Machine vision

C. elegans tracking in the low-magnification imaging stream

We used a combination of machine learning and motion detection for tracking *C. elegans* over the plate in the low-magnification imaging stream.

We trained WormCNN, a convolutional neural network (CNN) for making a binary prediction of whether a 1.58 mm x 1.58 mm region of interest (ROI) contains one or more worms. The structure of the WormCNN is shown in Fig. S5*A*. We curated a training dataset containing 43,568 ROI images (20 pixels x 20 pixels size), 50% worms, and 50% backgrounds (Fig. S5*B*). With a batch size of 24 and an SGD optimizer (momentum = 0.9), the model converged after 10 training epochs. The accuracy of the network is 97.23% on 10,784 test images (balanced test set, i.e., half worms and half backgrounds).

In addition to the results from the machine learning, we constructed a simple motion detector for identifying animals over the plate. The entire FOV is divided into multiple 0.63 mm x 0.63 mm ROIs. We took two frames Δt seconds apart, subtracted the pixel values, and summed up the pixel differences Δp within the ROI. If the Δp is higher than a threshold p_h, i.e., $\Delta p > p_h$, we consider the ROI containing animals. Values of Δt and Δp were calibrated on the animals of interest for the optimal performance.

Combining WormCNN and motion detection, the machine vision for *C. elegans* tracking functions in two modes: Finding mode and Tracking mode.

To rapidly identify animals over the plate, the Finding mode runs the motion detector over the entire FOV. Then it feeds the ROIs with the movement detected to WormCNN to check whether any animals are present in these ROIs. If so, the system sets the centroid coordinates of the ROIs to be the coordinates of the animals identified.

The Tracking mode tracks the animals using WormCNN, supplemented by subpixel processing. For an animal having a known coordinate $C_{n-1} = (X_{n-1}, Y_{n-1})$ for the frame $n-1$, the Tracking mode updates its coordinate for the frame n by a few steps.

First, it constructs a 2.53 mm x 2.53 mm rectangular tracking region, centering at C_{n-1}. Second, it splits the tracking region into multiple 1.58 mm x 1.58 mm ROIs separated by a constant step size (0.39 mm) in x and y directions. Each ROI is sent to WormCNN for generating a confidence score cs. The confidence score cs is the output of the final fully connected layer of WormCNN, indicating the likelihood of the input ROI containing any worms. Third, it calculates the centroid of the cs for all the ROIs in the tracking region and sets it to be the animal's coordinate in the frame n, i.e., $C_n = (X_n, Y_n)$. X_n and Y_n are given by

$$X_n = \frac{1}{CS}\sum cs_i x_i \, ; \; Y_n = \frac{1}{CS}\sum cs_i y_i$$

where cs_i is the confidence score of the i-th ROI; x_i, and y_i the x and y coordinates of the centroid of the i-th ROI; CS the sum of the confidence score for all the ROIs, i.e., $CS = \sum cs_i$.

In the equation above, x_i, and y_i are dependent on the coordinate of the animal in the frame $n-1$, i.e., C_{n-1}, the last known coordinate of the animal being tracked. Therefore, x_i and y_i can be written as $x_{n-1,i}$ and $y_{n-1,i}$ for indicating the temporal dependency. In short, the Tracking mode updates the coordinate of the animal for the current frame (X_n, Y_n) by its last seen coordinate (X_{n-1}, Y_{n-1}) by

$$(X_n, Y_n) = \left(\frac{1}{CS}\sum cs_i x_{n-1,i} \, , \frac{1}{CS}\sum cs_i y_{n-1,i}\right)$$

Using the Finding and the Tracking mode, WormPicker rapidly recognizes and moves individual animals to the high-magnification FOV, preparing for imaging and manipulation. This process involves a few steps.

First, the system runs the Finding mode to obtain the coordinate for a worm (X_w, Y_w). Then, the main gantry moves the image of the animal to the high-magnification FOV by translating by ΔX_g and ΔY_g in the x and y directions. ΔX_g and ΔY_g are given by

$$\Delta X_g = \frac{X_{hm} - X_w}{dp/dG}; \Delta Y_g = \frac{Y_{hm} - Y_w}{dp/dG}$$

where X_{hm} and Y_{hm} are the x and y coordinates of the high-magnification FOV; dp/dG the amount of the pixel shift corresponding to one unit movement in the main gantry. By making the gantry movement above, the animal is viewed in proximity to the high-magnification FOV.

The freely roaming animal can be kept centered at the high-magnification FOV by running the Tracking mode while the gantry is continuously making small-step jogging motions in x and y. The movements of the jogging in x and y are given by

$$dX_g = \frac{X_{hm} - X_{n,w}}{|X_{hm} - X_{n,w}|} S; dY_g = \frac{Y_{hm} - Y_{n,w}}{|Y_{hm} - Y_{n,w}|} S$$

where $X_{n,w}$ and $Y_{n,w}$ are the x and y coordinates of the worm in the current frame n, and S the predefined step size for the gantry jogging.

Pick tracking in the low-magnification imaging stream

To track the pick wire loop in the low-magnification imaging stream, we trained PickCNN, a CNN for determining whether a 1.26 mm x 1.26 mm ROI contains the pick tip. The backbone of PickCNN is the same as WormCNN (Fig. S5*A*). We curated a training dataset containing 872,550 ROI images (20 pixels x 20 pixels size), 50% containing the pick tip and 50% the backgrounds (Fig. S5*C*). The network was trained on a GPU (NVIDIA GeForce GTX 1660) using an SGD optimizer. The accuracy of the model is 99.36% on 50,400 test images (balanced test set, i.e., half pick tips and half backgrounds).

We applied PickCNN, supplemented with subpixel processing, to track the pick tip in real time. A 20 mm x 15 mm rectangular region centered at the low-magnification FOV is delineated

for tracking the pick tip inside the region. This tracking region is split into multiple 1.26 mm x 1.26 mm ROIs with a constant skip step (0.32 mm) in x and y directions. Each ROI is fed to PickCNN for generating a confidence score cs. The confidence score cs is the output of the final fully connected layer of PickCNN, indicating the likelihood of the ROI containing the pick tip.

Assuming there is only one picking arm visible, among all the ROIs, the one with the highest cs is the closest to the actual pick tip. We use (x_h, y_h) to denote the centroid coordinate of the ROI having the highest cs. By performing subpixel processing surrounding the (x_h, y_h), the coordinate of the pick tip (X_p, Y_p) can be inferred by

$$(X_p, Y_p) = \left(\frac{1}{CS}\sum cs_i x_i , \frac{1}{CS}\sum cs_i y_i\right); \forall(x_i, y_i): \sqrt{(x_i - x_h)^2 + (y_i - y_h)^2} < r$$

where cs_i is the confidence score of the i-th ROI; x_i, and y_i the x and y coordinates of the centroid of the i-th ROI; CS the sum of the confidence score for all the ROIs, i.e., $CS = \sum cs_i$. The equation above indicates that all the ROIs having the distance to (x_h, y_h) less than r are taken into the subpixel processing for determining the pick tip coordinate (X_p, Y_p). We set r = 1.89 mm.

We measured the precision of the pick tracking. The mean differences between the tracked position and the actual position are 94.9 μm (SD = 43.0 μm) in x and 284.5 μm (SD = 147.2 μm) in y (N = 225).

C. elegans segmentation in the high-magnification imaging stream

We trained a Worm-finding Mask Regional Convolutional Neural Network (1) (WorMRC), for pixel-wise segmentation for individual animals in the high-magnification FOV. We curated a training dataset containing 2,697 images (612 pixels x 512 pixels size and 1.88 mm x 1.57 mm FOV), over different developmental stages, ranging from L1 larvae to adult. For each training image, we manually labeled the contours for individual animals. WorMRC was adapted from a pretrained ResNet-50-FPN (1) backbone, and was trained at a GPU (NVIDIA GeForce GTX 1660 Ti) using an SGD optimizer (momentum = 0.9). The model converged after 10 training epochs. The network outputs bounding boxes for the objects that are likely to be the worm and generates

a contour for the object within the bounding box. WorMRC robustly segments individual animals over complex backgrounds (Fig. S5*D*). We plot the areas of the contours for individual animals (of mixed stages, L1s - adults) predicted by WorMRC against the results from manual segmentation in Fig. S5*E*. The WorMRC predicted contour area presents high consistency with the ground truth, R^2 = 0.972 (N = 690).

<u>Developmental stage determination</u>

The developmental stages of the animals in the high-magnification image were inferred by their lengths. WorMRC first generated contours for individual animals. The centerline of the individual contour was obtained by the methods previously reported (2). We used previously measured lengths for worms of various developmental stages (3) to obtain decision boundaries for approximately identifying the developmental stage: L1s are < 370 μm, L2s 370 – 500 μm, L3s 500 – 635 μm, L4s 635 – 920 μm, adults > 920 μm.

<u>Morphological analysis</u>

To perform the genetic cross between the *dpy-5* mutant and *dop-1p::GFP* transgenic (*Main Manuscript*, Fig. 3) we first developed an algorithm for identifying the Dumpy (Dpy) phenotype. The *dpy-5* mutant displays a shorter and stouter morphology than control animals. We measured the aspect ratio AR of the animal, $AR = \frac{A}{L^2}$, where A is the area of the contour segmented by WorMRC and L the length of the centerline of the worm contour (2). We used the automated system to record images for *dpy-5* mutants (CB61) and wild-type animals (N2) (N = 190).

Based on this dataset, we determined an AR-based threshold for identifying Dpy animals. The values of AR for individual Dpy and wild-type animals are plotted in Fig. S5*F*. With an 80%-20% train-test split, we constructed a one-dimensional support vector machine (SVM) to classify the Dpy and wild-type phenotypes based on the AR. The SVM-generated decision boundary gives 100% accuracy on both the training and the test sets (Fig. S5*F*). We used this threshold to classify future samples: if a worm has an AR greater than the threshold, then the worm is classified as Dpy; otherwise, it is classified as having a wild-type morphology.

Sex determination

C. elegans males and hermaphrodites display distinct tail morphologies, with a fan structure visible in the tails of males but not hermaphrodites. We trained a Sex-determining Mask Regional Convolutional Neural Network (1) (SexMRC), for determining the sexes of *C. elegans* based on the high-magnification images. We curated a training dataset containing 6,860 images (612 pixels x 512 pixels size and 1.88 mm x 1.57 mm FOV), for which the contours of heads, tails for hermaphrodites, and tails for males, were manually labeled. SexMRC was adapted from a pretrained ResNet-50-FPN (1) model and was trained with an SGD optimizer (momentum = 0.9) using a GPU (NVIDIA GeForce GTX 1660 Ti). The model converged after 7 training epochs. The model outputs bounding boxes for the objects that are likely to be *C. elegans* heads, tails for hermaphrodites, and males, as shown in Fig. S5*G*. The network identifies males with a 100% precision and an 85.71% sensitivity (recall) and identifies hermaphrodites with a 92.45% precision and a 92.45% sensitivity (recall) (N = 74).

Embryo detection

We trained an Embryo-finding Mask Regional Convolutional Neural Network (1) (EmbMRC), for identifying embryos in the high-magnification images. We curated a training dataset containing 454 images (612 pixels x 512 pixels size and 1.88 mm x 1.57 mm FOV), for which the contours of unhatched embryos were manually labeled. EmbMRC was adapted from a pretrained ResNet-50-FPN (1) backbone and was trained using an SGD optimizer at a GPU (NVIDIA GeForce GTX 1660 SUPER). The model converged after 10 training epochs. The model outputs bounding boxes for the objects that are likely to be the embryo and generates a contour for the object within the bounding box (Fig. S5*H*). Fig. S5*H* shows representative images along with the segmentation when a few, a medium number of, and a larger number of embryos are present in the FOV. The numbers of embryos counted by EmbMRC are highly consistent with the results from manual counting ($R^2 = 0.9772$, N = 124) (Fig. S5*I*).

Fluorescence phenotyping

For fluorescence phenotyping, the automated system moves to view individual worms in the high-magnification FOV and captures one bright field and one subsequent fluorescence frame. The bright field frame is delivered to WorMRC for obtaining contour segmentations for the animals. The fluorescent frame is binarized by an intensity threshold h_i and the bright spots obtained after the thresholding are filtered according to their sizes, for reducing noise. The bright spots with sizes larger than a threshold h_s are kept as true fluorescent spots; otherwise, they are discarded. Both h_i and h_s were calibrated on the fluorescent strains of interest. For each animal identified in the bright field image, we dilated the contour mask to compensate the potential mismatch between the bright field and fluorescent frames caused by the animal's free-roaming behaviors during the elapsed time (about 250 ms) for the frame acquisition. The animals cut off by the FOV boundaries were filtered out for fluorescence phenotyping, preventing false negatives. The parameters for fluorescence imaging and processing used for our experiments are given in Table S4.

For a moderately bright fluorescent strain (LX811), the algorithm classifies fluorescence phenotypes for individual animals of mixed stages with an accuracy of 97.16% (N = 211).

Automated verification of the transfer results

For the system to keep track of the working progress, we programmed the machine vision to verify whether the animal was successfully transferred to the destination. First, the robotic arm attempts to put down the animal in the high-magnification FOV, and it observes whether any worms can be found in the FOV using WorMRC. If so, the transfer is deemed a success; otherwise, continuing with the next step. Second, it observes whether any animals crawl out from the proximity of the spot where the worm was put down via the low-magnification FOV. If there are any such animals, the operation is deemed a success. The automated verification strategy achieves a 97.22% accuracy for verifying the results of the transfer attempts (N = 108).

Intermediate picking

Depending on the application, the automated system may be required to precisely transfer a single animal with some desired phenotypes and stray animals are strictly prohibited, for example, isolating F2s for identifying a transgenic integrant (*Main Manuscript*, Fig. 5). We programmed the automated system for those experiments to perform intermediate picking if multiple worms or/and unhatched embryos are identified in the high-magnification FOV.

The system first inspects all the animals in the image, if none of them carries the desired phenotypes, the system inspects other animals; if at least one carries the desired phenotypes, the robotic arm picks up the target animal, possibly along with surrounding ones. Then, the system puts the animals down onto a fresh intermediate plate and waits a few seconds allowing the worms to move. The system then picks up the single animal with the desired phenotypes.

Theory of the genetic mapping experiment

Using the automated methods, we genetically mapped an RFP transgene *vsIs33 [dop-3p::RFP]* using classic genetics. We tested the linkage of *vsIs33* to different chromosomes by setting up genetic crosses between the strain of interest (LX811) and a set of genetic balancers labeled by GFP markers.

The first genetic balancer strain we used was JK2810, carrying a *hT2* (4) reciprocal translocation in I and III, for determining linkage to I and III. As shown in Fig. S8, WormPicker crossed JK2810 males with LX811 hermaphrodites and picked out F1 cross-progenies that were both Red and Green. According to the theory, the F2 self-progenies descending from these double-fluorescent F1s would display a segregation pattern for the Red and the Green, as shown in the Punnett squares (Fig. S8). If *vsIs33* is linked to neither I nor III, the theory predicts 75% of the nonGreen animals to be Red, i.e., P (Red | nonGreen) = 0.75 (Fig. S8*A*); while if *vsIs33* is linked to either I or III, 100% of the nonGreen would be Red, i.e., P (Red | nonGreen) = 1 (Fig. S8*B*).

We determined the linkage of *vsIs33* to other autosomes using the same scheme. The automated system generated crosses between LX811 and other GFP-labeled genetic balancers, picked double-fluorescent F1 cross-progeny, and screened for the segregation pattern for the Red and the Green in F2s. These balancer strains include VC170, carrying a *mIn1* (5) inversion in II (Fig. S9), CGC34, carrying an *eT1* (6, 7) reciprocal translocation in III and V (Fig. S10), JK2958, carrying a *nT1* (8-10) reciprocal translocation in IV and V (Fig. S11). For all the autosomal genetic balancers, the theory predicts 75% of the nonGreen F2s would be Red if unsuccessfully balanced, and 100% if successfully balanced.

To test the linkage of *vsIs33* to the X chromosome requires a different scheme (Fig. S12). WormPicker crossed wild-type males harboring a dominant extrachromosomal transgene *qnEx615[myo-2p::GFP]* (NQ1155) with LX811 hermaphrodites, and picked out double-fluorescent F1 males, which were subsequently crossed with wild-type (N2) hermaphrodites. The theory predicts that none of F2 males would be Red if the transgene of interest is X-linked, and 50% if not X-linked.

For each linkage test for the autosomes described above, WormPicker screened over the F2s and obtained the numbers of the nonGreen animals that were Red, N_{A1}, and nonRed, N_{A2}, and the total numbers of the nonGreen animals found, $N = N_{A1} + N_{A2}$. As shown in Fig. S13*A,* the red bars represent the observed percentages of the Red among the nonGreen animals, $p_{A1} = \frac{N_{A1}}{N}$, while the gray bars the percentages of the nonRed among the nonGreen animals, $p_{A2} = \frac{N_{A2}}{N}$. The error bars indicate the standard deviation SD_A of the observed percentages under the autosome-unlinked assumption, where $SD_A = \sqrt{\frac{p_{AUL}(1-p_{AUL})}{N}}, p_{AUL} = 0.75$.

Similarly, for testing the X-linkage, the automated system screened over the F2s and obtained the number of males that were Red, N_{X1}, and nonRed, N_{X2}, and the total number of males found $N = N_{X1} + N_{X2}$. As shown in Fig. S13*B*, the red bar represents the observed percentage of Red among males, $p_{X1} = \frac{N_{X1}}{N}$, while the gray bar the percentage of nonRed among

males, $p_{X2} = \frac{N_{X2}}{N}$. The error bars indicate the standard deviation SD_X of the observed percentages under the X-unlink assumption, where $SD_X = \sqrt{\frac{p_{XUL}(1-p_{XUL})}{N}}, p_{XUL} = 0.5$.

To evaluate the linkage strength, we developed a Linkage Index to quantify the similarity between the theoretical and the observed link (Fig. S13*C*). To derive the Linkage Index, we first vectorize the link pattern in theory $\boldsymbol{W}_L$, the unlink pattern in theory $\boldsymbol{W}_{UL}$, and the observed pattern $\boldsymbol{W}_{obs}$:

$$\boldsymbol{W}_L = \begin{bmatrix} p_L \\ 1 - p_L \end{bmatrix}, \boldsymbol{W}_{UL} = \begin{bmatrix} p_{UL} \\ 1 - p_{UL} \end{bmatrix}, \boldsymbol{W}_{obs} = \begin{bmatrix} N_1 \\ N_2 \end{bmatrix}$$

For the autosome-linkage test, p_L and p_{UL} are the fractions of the nonGreen animals that would be Red for the link and the unlink patterns in theory, i.e., $p_L = 1, p_{UL} = 0.75$; N_1 and N_2 are the observed count numbers of the nonGreen animals that were Red and nonRed. For the X linkage test, p_L and p_{UL} are the fraction of the males that would be Red for the X-link and X-unlink patterns in theory, i.e., $p_L = 0, p_{UL} = 0.5$; N_1 and N_2 are the observed count numbers of males that were Red and nonRed.

Let $\boldsymbol{e}_L$ and $\boldsymbol{e}_{UL}$ be the unit vectors pointing to the same directions as $\boldsymbol{W}_L$ and $\boldsymbol{W}_{UL}$, i.e.,

$$\boldsymbol{e}_L = \frac{\boldsymbol{W}_L}{\|\boldsymbol{W}_L\|}, \boldsymbol{e}_{UL} = \frac{\boldsymbol{W}_{UL}}{\|\boldsymbol{W}_{UL}\|}$$

(|| || denotes the L2-norm of the vector). Then we set $\boldsymbol{e}_L$ and $\boldsymbol{e}_{UL}$ as the basis vectors and $\boldsymbol{W}_{obs}$ can be expressed in terms of the basis

$$\boldsymbol{W}_{obs} = v_1\boldsymbol{e}_L + v_2\boldsymbol{e}_{UL} = [\boldsymbol{e}_L, \boldsymbol{e}_{UL}]\boldsymbol{v}_{obs}$$

where $\boldsymbol{v}_{obs} = \begin{bmatrix} v_1 \\ v_2 \end{bmatrix}$ is the coordinate of $\boldsymbol{W}_{obs}$ with respect to the basis $\boldsymbol{e}_L$ and $\boldsymbol{e}_{UL}$. $\boldsymbol{v}_{obs}$ can be obtained by

$$\boldsymbol{v}_{obs} = [\boldsymbol{e}_L, \boldsymbol{e}_{UL}]^{-1}\boldsymbol{W}_{obs}$$

where $[\quad]^{-1}$ denotes the matrix inverse. The coordinates of the basis vectors themselves are $\boldsymbol{v}_L = [\boldsymbol{e}_L, \boldsymbol{e}_{UL}]^{-1}\boldsymbol{e}_L = \begin{bmatrix}1\\0\end{bmatrix}$ for $\boldsymbol{e}_L$ and $\boldsymbol{v}_{UL} = [\boldsymbol{e}_L, \boldsymbol{e}_{UL}]^{-1}\boldsymbol{e}_{UL} = \begin{bmatrix}0\\1\end{bmatrix}$ for $\boldsymbol{e}_{UL}$.

We define the Linkage Index $Lidx$ as the cosine similarity between the $\boldsymbol{v}_{obs}$ and $\boldsymbol{v}_L$:

$$Lidx = cos\,\theta = \frac{\langle \boldsymbol{v}_{obs}, \boldsymbol{v}_L \rangle}{\|\boldsymbol{v}_{obs}\| \cdot \|\boldsymbol{v}_L\|}$$

where θ is the angle formed by the $\boldsymbol{v}_{obs}$ and $\boldsymbol{v}_L$ (Fig. S13*C*) and $\langle\quad\rangle$ denotes the vector inner product. If the observed pattern is close to the link pattern, then $\theta \to 0$, yielding $Lidx \to 1$, implying a strong linkage to the chromosomes of interest. If, on the other hand, the observed pattern is similar to the unlink pattern, the $\theta \to 90°$, yielding $Lidx \to 0$, implying a weak linkage. Note that we cannot draw any conclusions when $Lidx \to -1$ because it suggests that the observed pattern is far deviated from both the link and the unlink patterns.

The observed Linkage Indices for *vsIs33* over different chromosomes are shown in Fig. S13*D*. We found that *vsIs33* displays strong linkage to either III or V, and to either IV or V, suggesting the transgene of interest is linked to V.

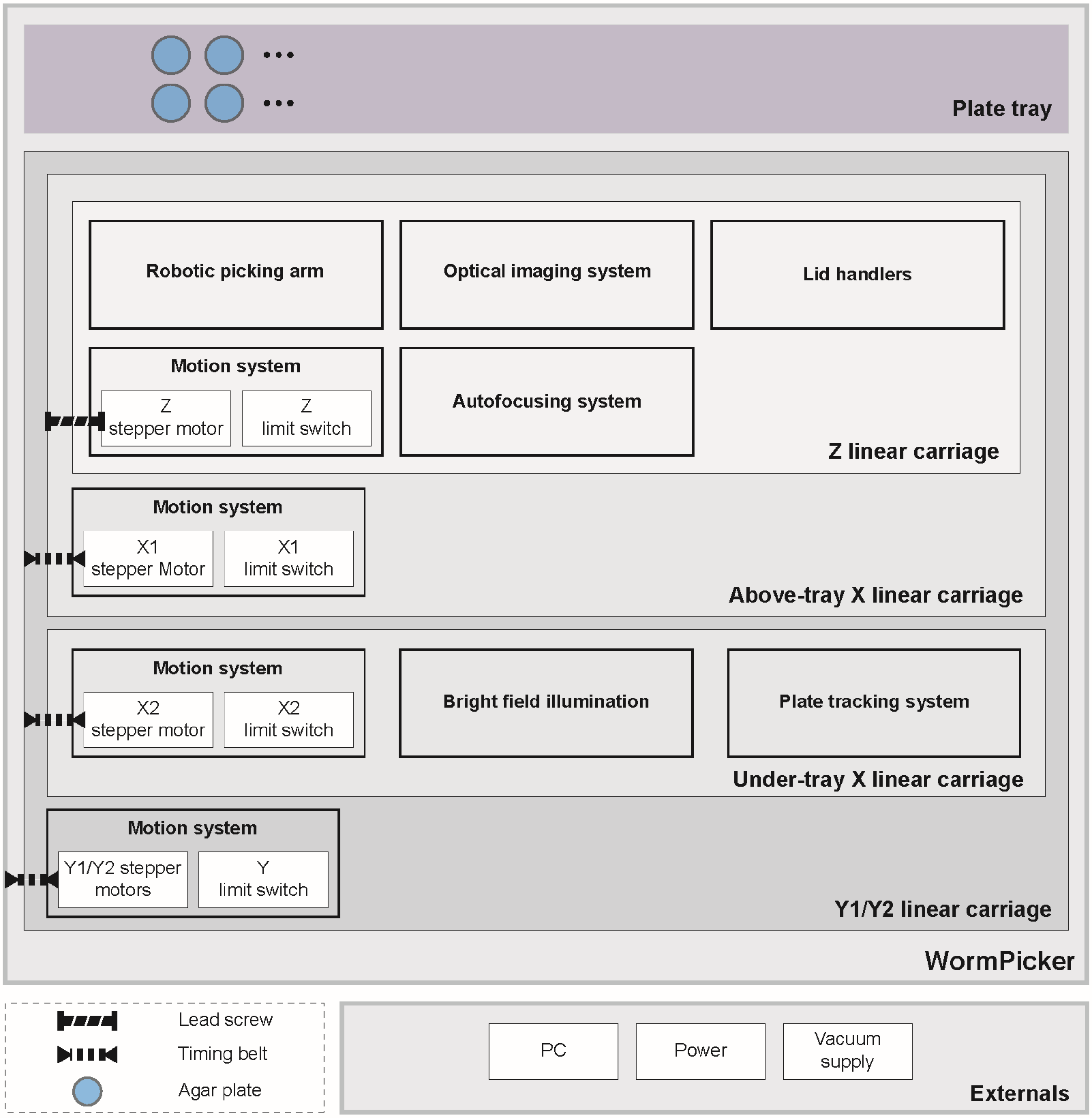

Fig. S1. WormPicker hardware architecture. WormPicker is composed of a plate tray platform housing an array of agar substrates and linear carriage assemblies built in Cartesian coordinates, i.e., X, Y, and Z. The Z linear carriage motorizes imaging and manipulation assemblies, including a robotic picking arm, an optical imaging system, two lid handlers, and an image autofocusing system. The under-tray X linear carriage motorizes supplementary tools, including a bright field illumination and a plate tracking module.

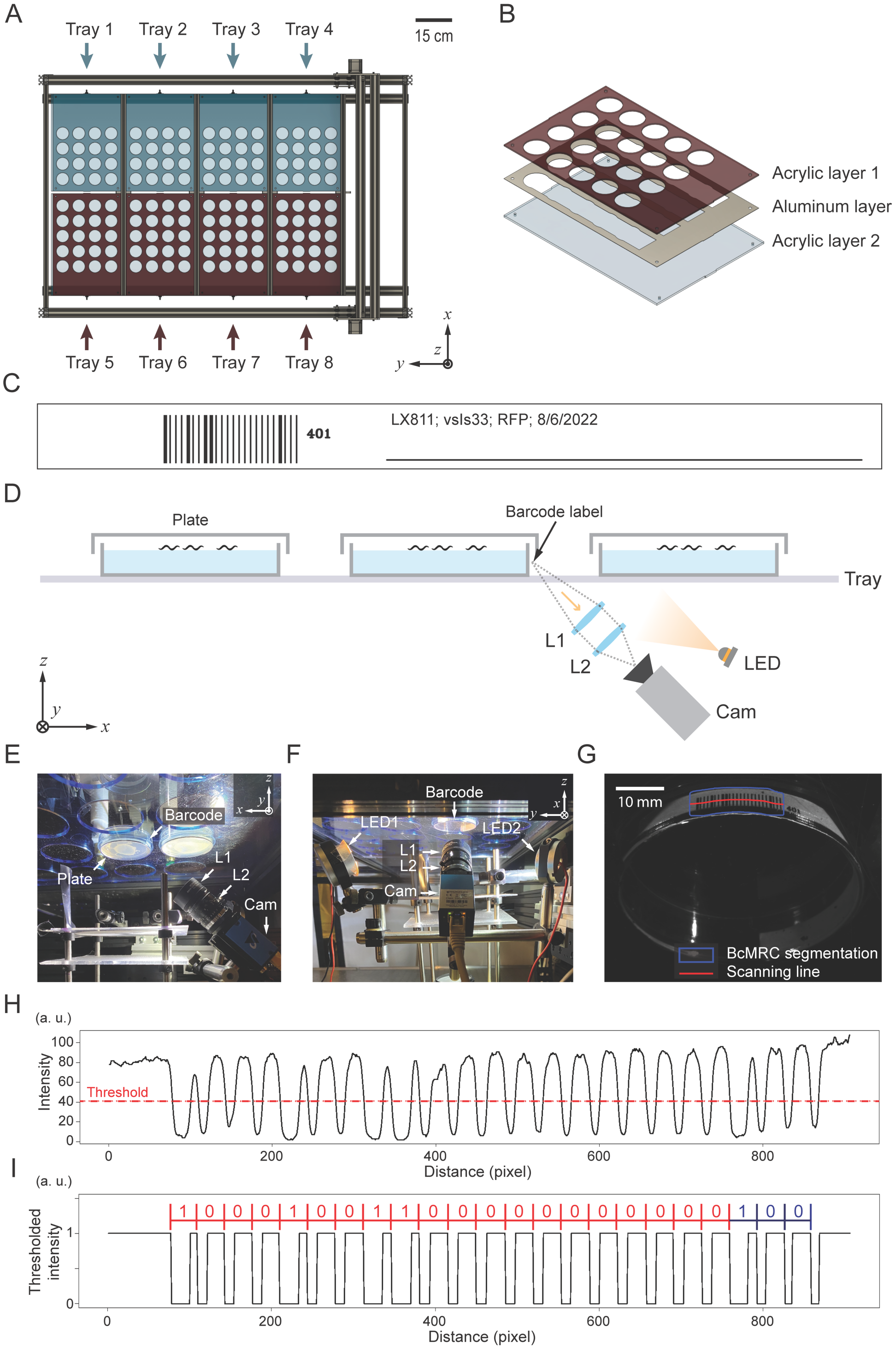
A
Tray 1
Tray 2
Tray 3
Tray 4
15 cm
Tray 5
Tray 6
Tray 7
Tray 8
x
z
y
B
Acrylic layer 1
Aluminum layer
Acrylic layer 2
C
401
LX811; vsIs33; RFP; 8/6/2022
D
Plate
Barcode label
Tray
L1
L2
LED
Cam
z
y
x
E
Barcode
Plate
L1
L2
Cam
F
Barcode
LED1
LED2
L1
L2
Cam
G
10 mm
BcMRC segmentation
Scanning line
H
(a. u.)
Intensity
Threshold
Distance (pixel)
I
(a. u.)
Thresholded intensity
1 0 0 0 1 0 0 1 1 0 0 0 0 0 0 0 0 0 0 0 1 0 0
Distance (pixel)

Fig. S2. WormPicker plate tray platform and plate tracking assemblies. *(A)* Top view. The platform is composed of 8 easy slide-in plate trays. *(B)* Each slide-in plate tray consists of two transparent laser-cut acrylics sandwiching an aluminum layer. *(C)* A barcode label. *(D)* Machine vision system for barcode reading. *(E* and *F)* Front and side view of the barcode-reading apparatus. *(G)* Camera-captured image of the barcode label with BcMRC segmentation and a scanning line. *(H)* Profile of pixel intensity along the scanning line drawn in *(G)*. Red dashed line: threshold for binarizing the intensity profile. *(I)* The binarized intensity profile of *(H)*. Red: decoded 20-bit number; Blue: 3-bit checksum.

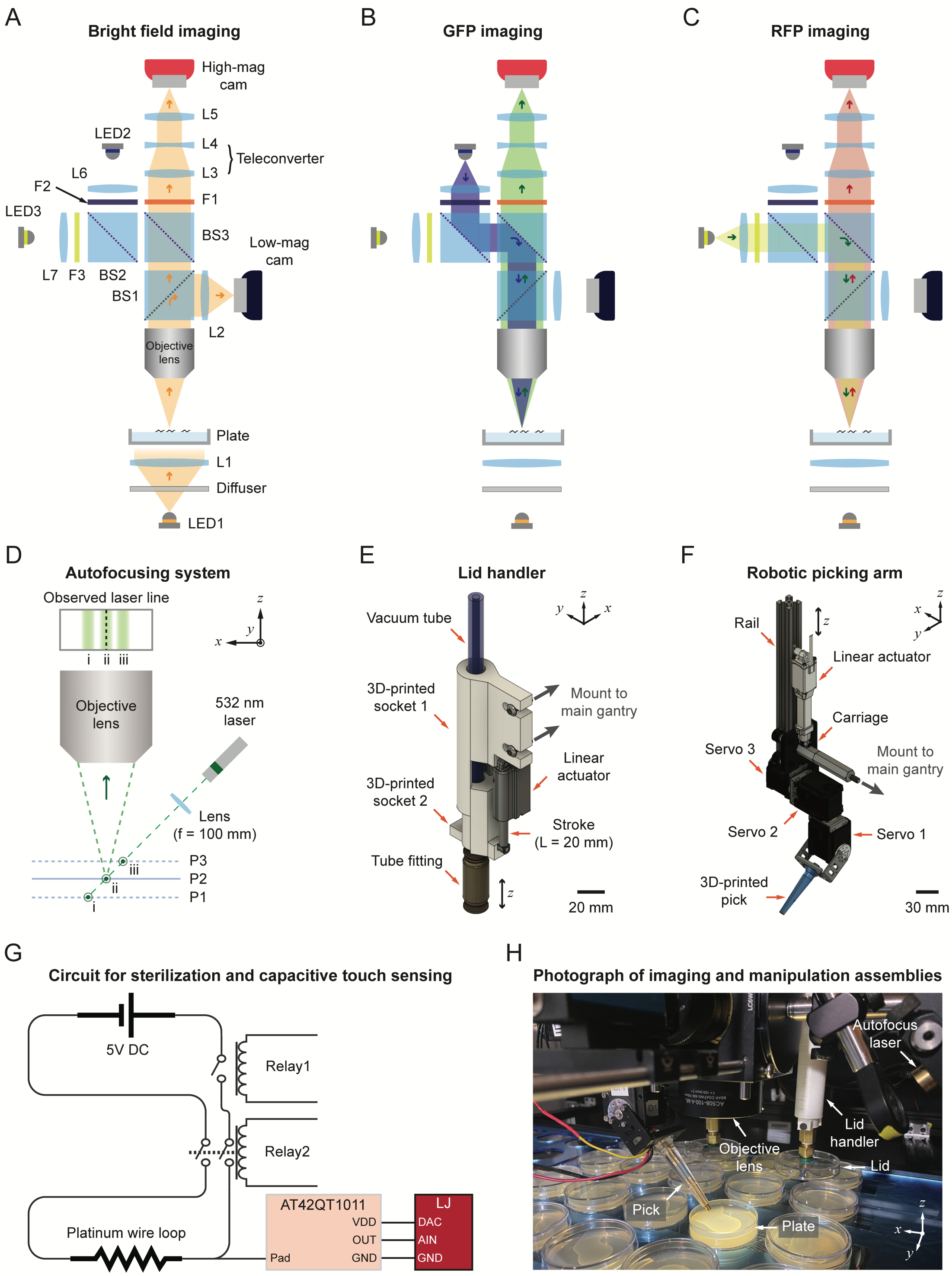
A
Bright field imaging
High-mag cam
L5
LED2
L4
Teleconverter
L6
L3
F2
F1
LED3
BS3
Low-mag cam
L7 F3 BS2
BS1
L2
Objective lens
Plate
L1
Diffuser
LED1
B
GFP imaging
C
RFP imaging
D
Autofocusing system
Observed laser line
i ii iii
Objective lens
532 nm laser
Lens (f = 100 mm)
P3
P2
P1
E
Lid handler
Vacuum tube
3D-printed socket 1
Mount to main gantry
Linear actuator
3D-printed socket 2
Stroke (L = 20 mm)
Tube fitting
20 mm
F
Robotic picking arm
Rail
Linear actuator
Carriage
Servo 3
Mount to main gantry
Servo 2
Servo 1
3D-printed pick
30 mm
G
Circuit for sterilization and capacitive touch sensing
5V DC
Relay1
Relay2
AT42QT1011
VDD
OUT
Pad
GND
LJ
DAC
AIN
GND
Platinum wire loop
H
Photograph of imaging and manipulation assemblies
Autofocus laser
Lid handler
Objective lens
Lid
Pick
Plate

Fig. S3. WormPicker imaging and manipulation assemblies. *(A-C)* Schematic of *(A)* bright field, *(B)* GFP, and *(C)* RFP imaging. BS1: beamsplitter (transmission : reflection = 90% : 10%). BS2 and 3: long-pass and dual-band dichroic beamsplitters. F1: dual-band emission filter. F2 and 3: excitation filters for GFP and RFP. L1: Fresnel lens. L2: machine vision lens. L3 and 4: teleconverter lens pair (f = 300 mm and -100 mm). L5: tube lens. L6 and 7: collimating lenses. LED1-3: white, 470 nm, and 565 nm LED. Objective lens: achromatic doublet lens (f = 100 mm). *(D)* Schematic of autofocusing system. P1-3: different positions of an agar surface, where P2 is the focus position. i-iii: positions of a laser line projected to the agar surface at different planes, i.e., P1-3. Dashed line: pre-calibrated optimal position. *(E)* Schematic of lid handler. *(F)* Schematic of robotic picking arm. *(G)* Electric circuit for pick sterilization and capacitive touch sensing. AT42QT1011: capacitive touch sensor; LJ: LabJack data acquisition device. *(H)* Photograph of imaging and manipulation assemblies.

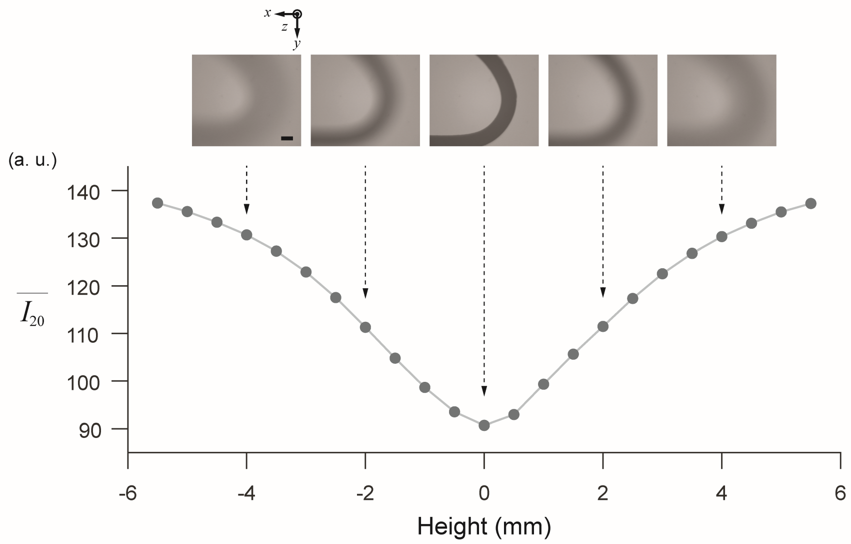

Fig. S4. Mean intensity of the top 20% darkest pixels in the high-magnification FOV ($\overline{I_{20}}$) for the pick tip at different heights relative to the optimal focus. Above: pick tip images. Scale bar: 200 μm.

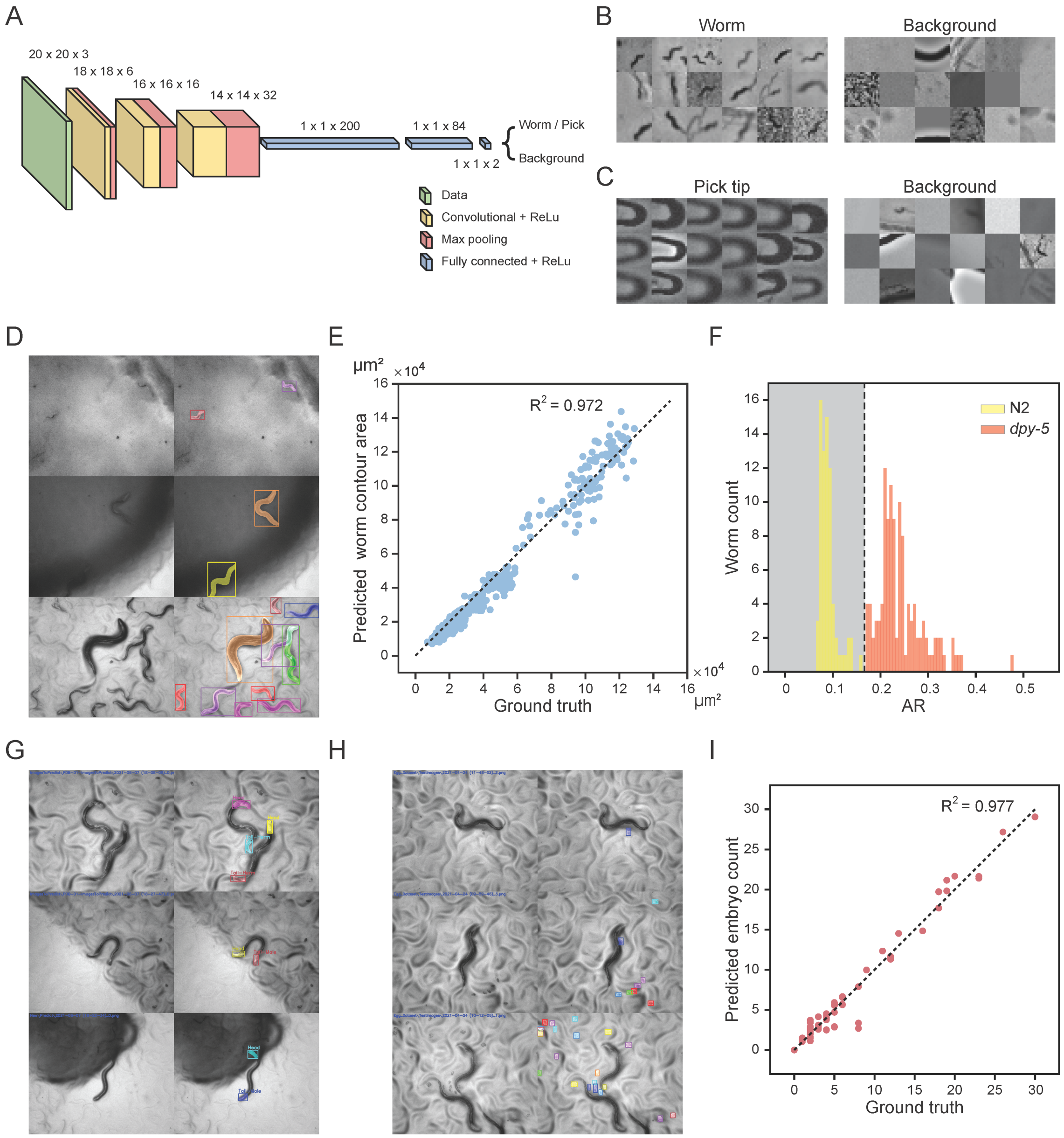
A
20 x 20 x 3
18 x 18 x 6
16 x 16 x 16
14 x 14 x 32
1 x 1 x 200
1 x 1 x 84
1 x 1 x 2
Worm / Pick
Background
Data
Convolutional + ReLu
Max pooling
Fully connected + ReLu
B
Worm
Background
C
Pick tip
Background
D
E
μm²
×10⁴
R² = 0.972
Predicted worm contour area
Ground truth
μm²
F
N2
dpy-5
Worm count
AR
G
H
I
R² = 0.977
Predicted embryo count
Ground truth

Fig. S5. Machine vision analysis. *(A)* Structure of WormCNN and PickCNN. *(B)* Representative images of worm (left) and background (right) for WormCNN training. FOV: 1.58 mm x 1.58 mm. *(C)* Representative images of pick tip (left) and background (right) for PickCNN training. FOV: 1.26 mm x 1.26 mm. *(D)* Worm segmentations by WorMRC (left: raw images; right: segmentations). FOV: 1.88 x 1.57 mm. Individual worms are labeled with different colors. *(E)* Scatter plot of areas of individual worm contours (of mixed stages, L1- Adult) segmented by WorMRC versus ground truth (N = 690). Dashed line: $\boldsymbol{y} = \boldsymbol{x}$. *(F)* Histogram of aspect ratios (ARs) of wild-type (N2) and Dpy (*dpy-5*) animals (N = 190). Black dashed line: decision boundary for classifying wild-type (gray region) and dumpy morphology (white region). *(G)* Sex determination by SexMRC (left: raw images; right: segmentations). Individual heads and tails are labeled with different colors. Recognized types of the tails are indicated by the text. *(H)* Embryo detection by EmbMRC (left: raw images; right: segmentations). Individual embryos or clusters of embryos are labeled with different colors. *(I)* Scatter plot of numbers of embryos detected by EmbMRC versus ground truth (N = 124). Dashed line: $\boldsymbol{y} = \boldsymbol{x}$.

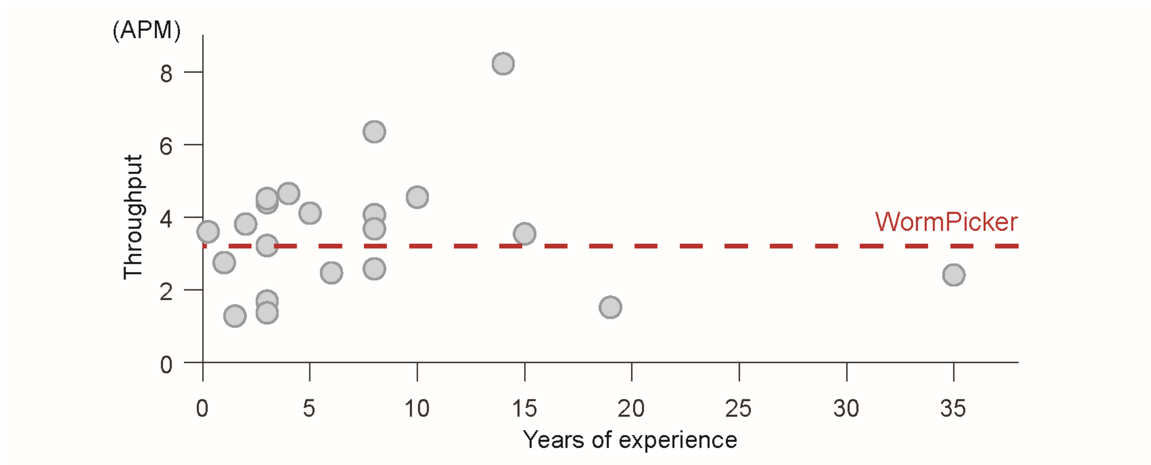

Fig. S6. Scatter plot of picking throughput (unit: Animals per Minute, APM) versus years of experience for a fluorescent animal sorting task performed by a group of researchers (N = 21). Mean and median years of experience: 7.61 and 5 years. Mean, median, and standard deviation (SD) of manual throughput: 3.56, 3.60, and 1.67 APM. Dashed line: automated throughput, 3.21 APM (SD = 0.66 APM, N = 38).

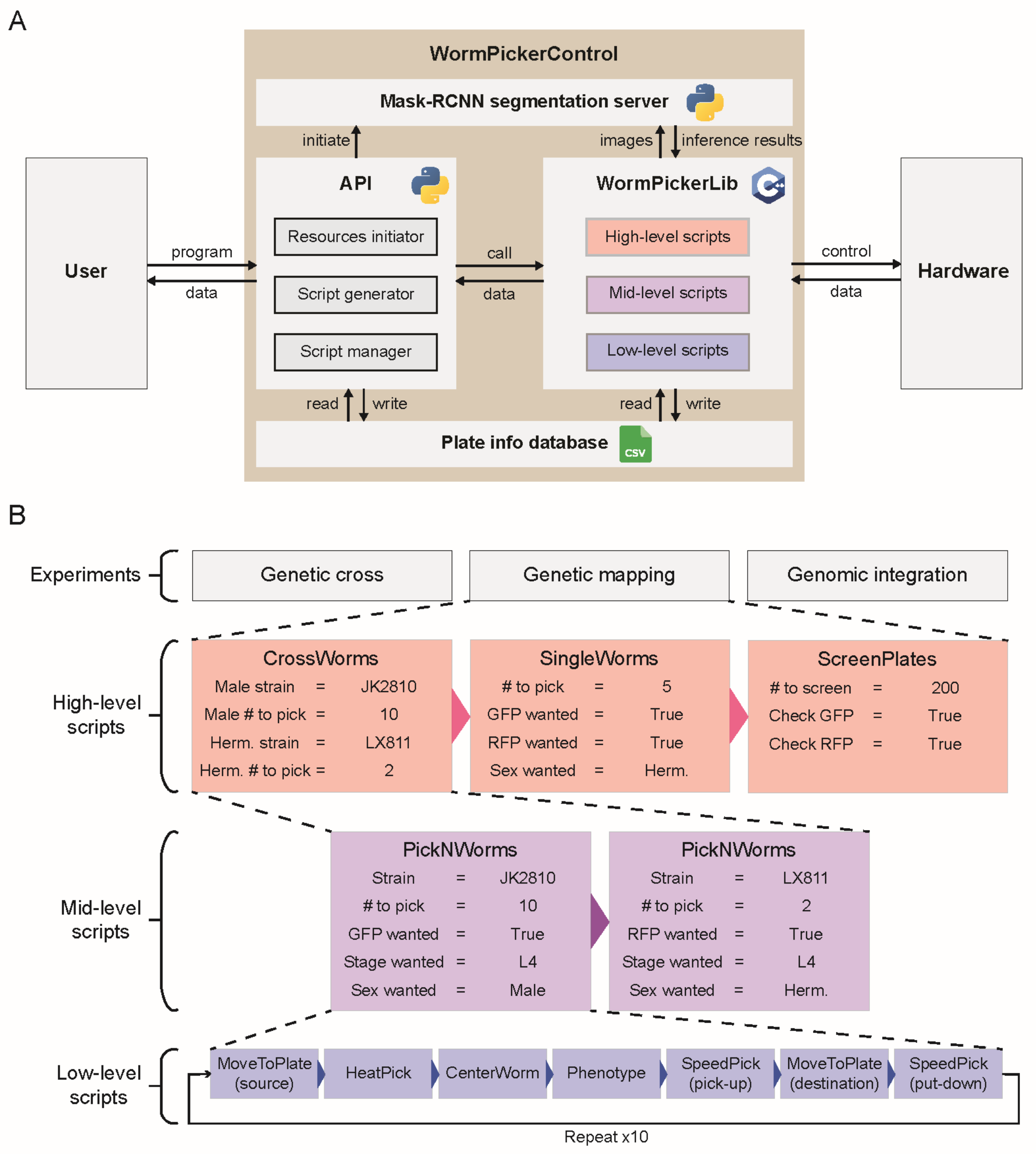

Fig. S7. WormPickerControl and WormPickerLib. *(A)* Schematic of WormPickerControl. WormPickerControl is composed of four modules, including an Application Programming Interface (API), WormPickerLib (a library of source scripts), a Mask-RCNN segmentation server, and a database cataloging plate information. *(B)* Schematic of WormPickerLib, demonstrating its hierarchical structure by taking the genetic mapping experiment as an example (cross JK2810 with LX811, as shown in *Main Manuscript*, Fig. 4*C*).

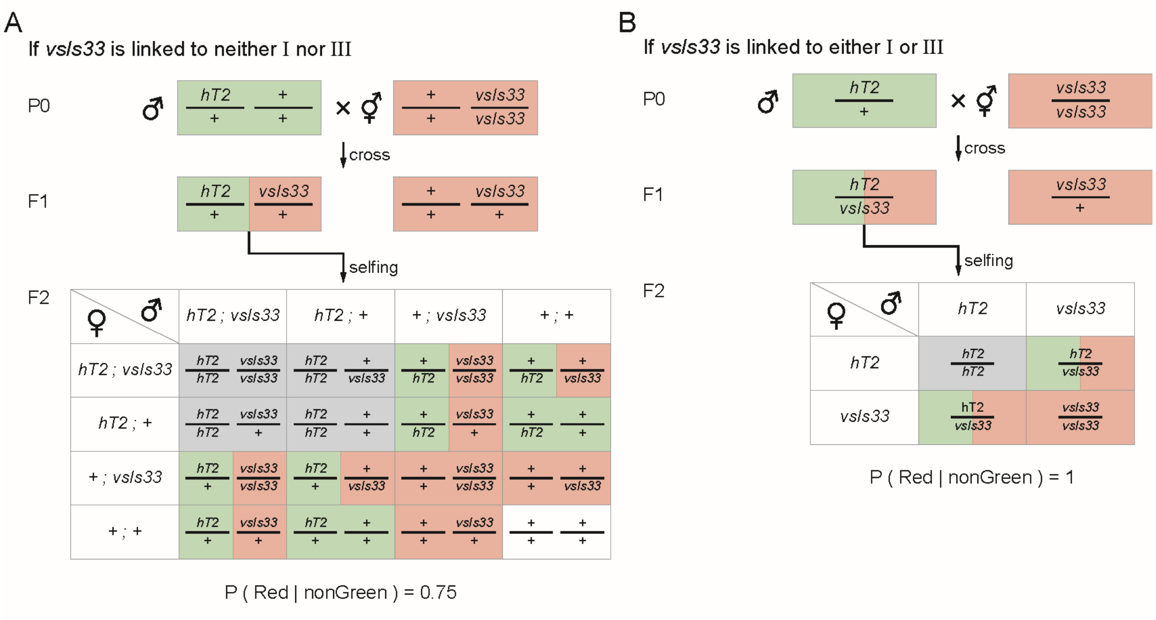

Fig. S8. Theory of the I/III-linkage test. *(A)* The Punnett square showing the F2s to be observed if *vsIs33* is linked to neither I nor III. *hT2* is homozygous lethal. *(B)* The Punnett square showing the F2s to be observed if *vsIs33* is linked to either I or III. Green: green fluorescent animal. Red: red fluorescent animal. Gray: inviable animal.

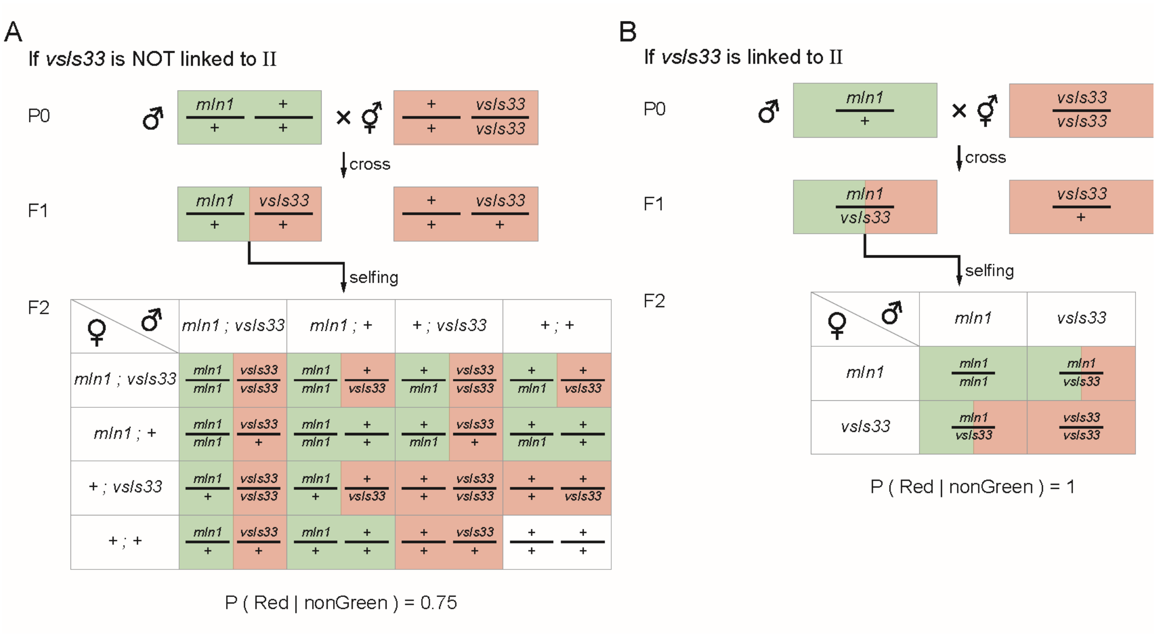

Fig. S9. Theory of the II-linkage test. *(A)* The Punnett square showing the F2s to be observed if *vsIs33* is not linked to II. *(B)* The Punnett square showing the F2s to be observed if *vsIs33* is linked to II. The color code is the same as in Fig. S8.

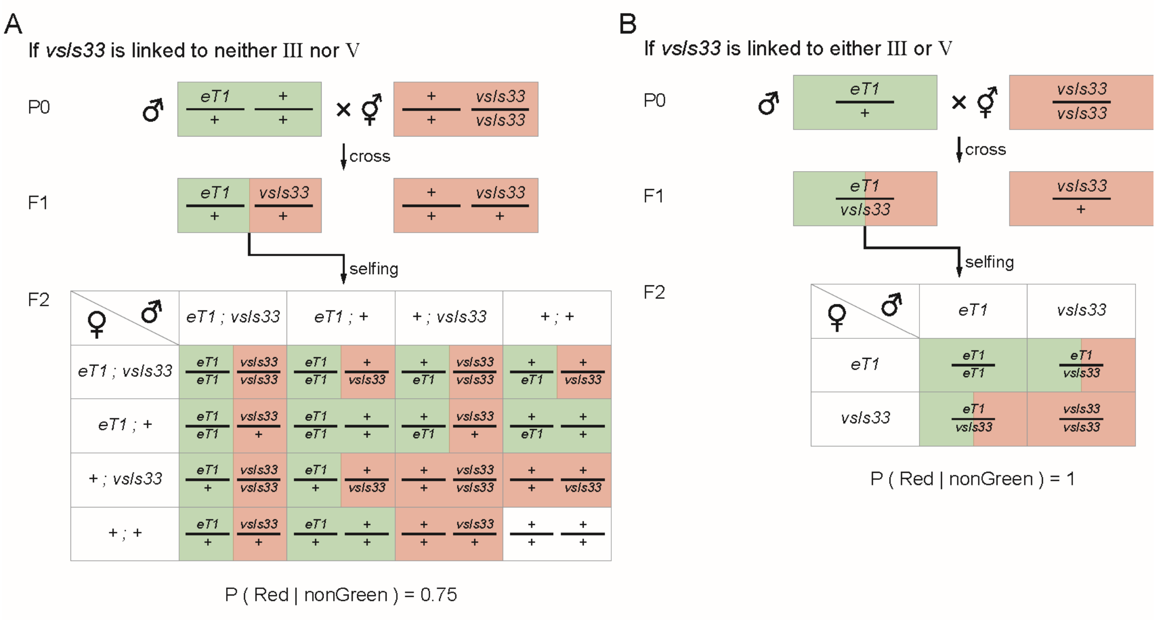

Fig. S10. Theory of the III/V-linkage test. *(A)* The Punnett square showing the F2s to be observed if *vsIs33* is linked to neither III nor V. *(B)* The Punnett square showing the F2s to be observed if *vsIs33* is linked to either III or V. The color code is the same as in Fig. S8.

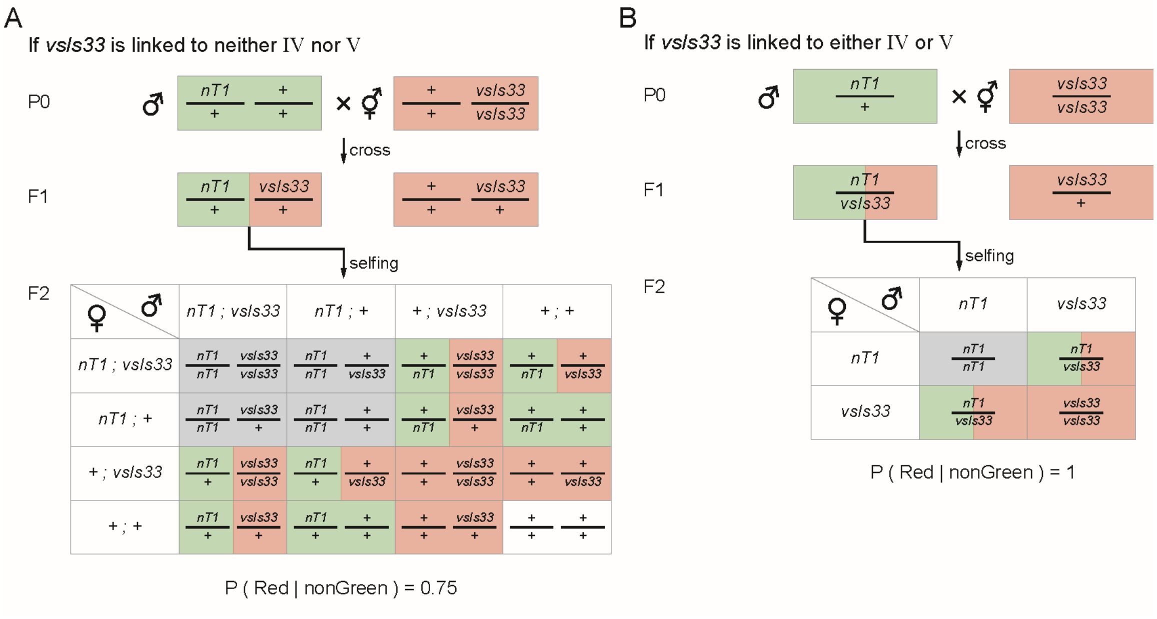

Fig. S11. Theory of the IV/V-linkage test. *(A)* The Punnett square showing the F2s to be observed if *vsIs33* is linked to neither IV nor V. *nT1* is homozygous inviable. *(B)* The Punnett square showing the F2s to be observed if *vsIs33* is linked to either III or V. The color code is the same as in Fig. S8.

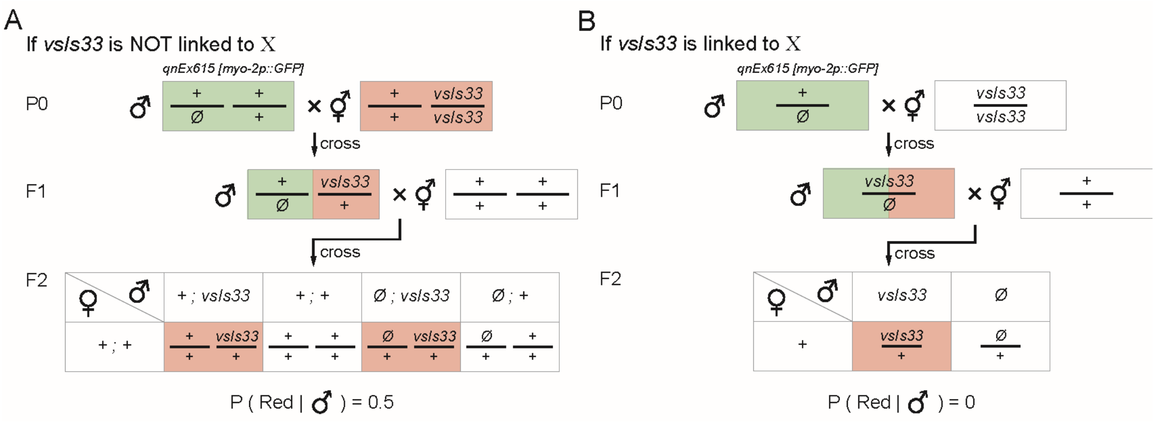

Fig. S12. Theory of the X-linkage test. *(A)* The Punnett square showing the F2s to be observed if *vsIs33* is not linked to X. *(B)* The Punnett square showing the F2s to be observed if *vsIs33* is linked to X. The color code is the same as in Fig. S8.

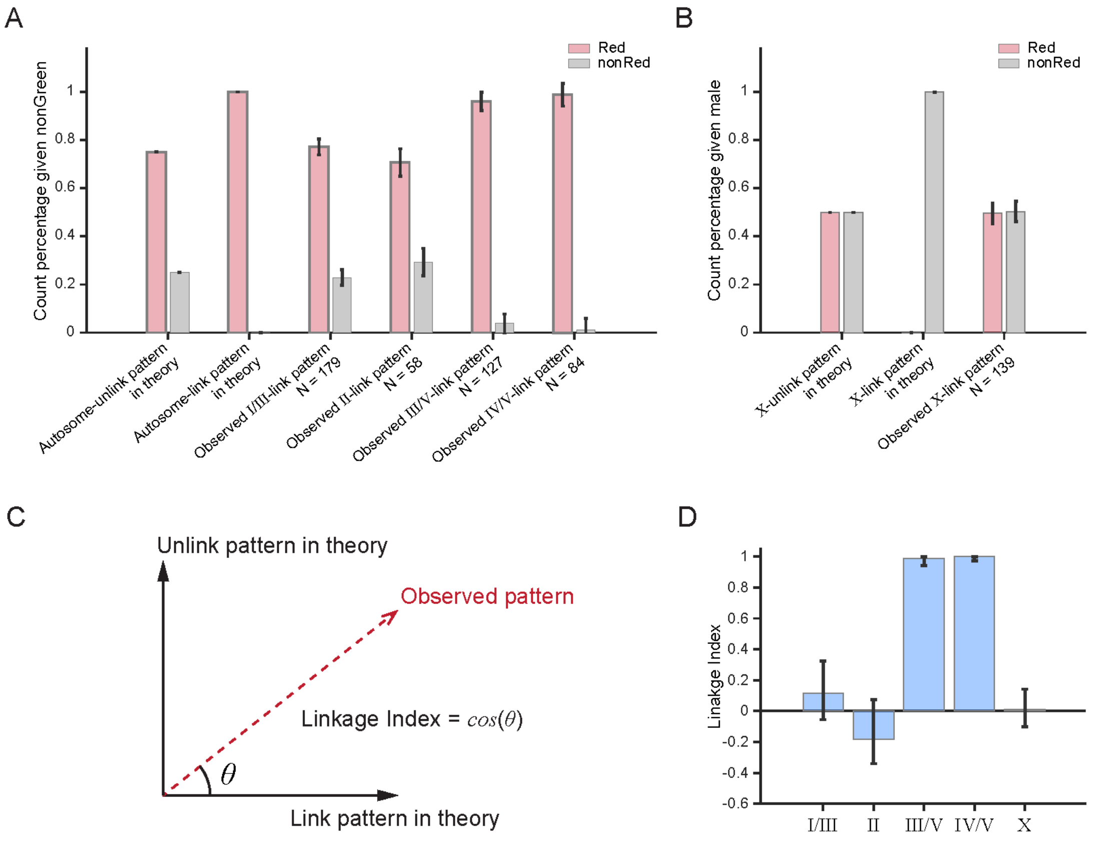

Fig. S13. Data of the linkage test for *vsIs33*. *(A)* The theory predicted and the observed count percentages of Red and nonRed among the nonGreen F2s for the autosome-linkage test. Error bar: standard deviation of the observed percentages under the autosome-unlink assumption. *(B)* The theory predicted and the observed count percentages of Red and nonRed among the F2 males for the X-linkage test. Error bar: standard deviation of the observed percentages under the X-unlink assumption. *(C)* Graphical representation of Linkage Index. *(D)* Observed Linkage Indices for *vsIs33* over different chromosomes. Error bar: standard deviation of the observed Linkage Indices under the unlink assumption.

Table S1. Viability data for the animals 24 hours after the WormPicker and manual picking. P_a, P_d, and P_e denote the percentage of the animal alive, dead, and escaped; N denotes the number of animals picked.

Genotype	Phenotype	Stage	Automated picking				Manual picking			
			P_a	P_d	P_e	N	P_a	P_d	P_e	N
N2	Wild type	L1	90.7	-	-	54	98	0	2	50
		L2	92.8	-	-	83	98	0	2	50
		L3	96.7	0	3.3	90	98	0	2	50
		L4	98.6	0	1.4	145	100	0	0	50
		Day 1 adult	94.3	0	5.7	105	100	0	0	60
		Day 5 adult	96.4	3.6	0	84	97.1	2.9	0	35
lin-15	Multivulva	Adult	81.5	17	1.5	65	92	8	0	50
rol-6	Roller	L4 - Adult	98	0	2	50	100	0	0	61
unc-13	Uncoordinated, paralyzed	L3 - Adult	100	0	0	53	100	0	0	50
lpr-1	Fragile cuticles	L4 - Adult	98.1	0	1.9	54	98	2	0	50

Table S2. Success rates for WormPicker picking up and putting down *C. elegans*. $\boldsymbol{P_s}$ denotes the percentage of the successful attempts; $\boldsymbol{N}$ denotes the total number of the attempts made.

Plate type	Genotype	Phenotype	Stage	Pick-up		Put-down	
				P_s	N	P_s	N
Seeded	N2	Wild type	L3	92.7	41	100	38
			L4	95.8	71	95.7	69
			Day 1 adult	90.2	112	96.1	103
			Day 5 adult	97.9	47	89.4	47
	lin-15	Multivulva	Adult	91.4	70	98.5	65
	rol-6	Roller	L4 - Adult	93.3	45	93	43
	unc-13	Uncoordinated, paralyzed	L3 - Adult	87.2	39	97.1	34
	lpr-1	Fragile cuticles	L4 - Adult	98	51	92.3	52
Unseeded	N2	Wild type	L4	92.6	54	98	51

Table S3. WormPicker key components list.

Part	Item number (Vendor)	Qty	Unit cost (USD)	Amount (USD)
**Motion system**				
XYZ moving stage - 60" x 40"	2415-Bundle (OpenBuilds)	1	1986	1986
**Robotic picking arm**				
3D-printed pick	(Xometry)	1	78	78
90% platinum, 10% iridium wire - 5cm	PT-9010 (Tritech Research)	1	7	7
Capacitive touch sensor	AT42QT1011 (SparkFun)	1	7	7
Carriage	1185-Set (OpenBuilds)	1	35	35
Contact pin 14-18AWG crimp	Newark Electronics	2	1	2
Linear actuator	S20-30-38-B (Actuonix)	1	80	80
Linear rail, L = 250 mm	280-LP (OpenBuilds)	1	3	3
Servo motor	RO-902-0067-000 (Trossen Robotics)	3	260	780
**Optical imaging system**				
Collimating lens - 470 nm LED	SFM80 (Thorlabs)	1	965	965
Collimating lens - 565 nm LED	ACL2520U-A (Thorlabs)	1	33	33
Beamsplitter (Ø2" 10:90 (R:T))	BSN16 (Thorlabs)	1	220	220
Diffuser	Rock Hard Plastics frosted acrylic sheet (Amazon)	1	10	10
Dual-band dichroic beamsplitter	59022bs (Chroma)	1	325	325
Dual-band emission filter	59022m (Chroma)	1	350	350
Excitation filter - GFP	XF2015 (Omega Optical)	1	200	200
Excitation filter - RFP	65-705 (Edmund Optics)	1	160	160
Fresnel lens	46-614 (Edmund optics)	1	89	89
High-mag camera	CS505MU (Thorlabs)	1	2713	2713

LED - 470 nm	M470L5 (Thorlabs)	1	239	239
LED - 565 nm	M565L3 (Thorlabs)	1	252	252
Long-pass dichroic beamsplitter	XF2010 (Omega Optical)	1	200	200
Low-mag camera	DMK33GP031 or DMK33GR0521(Imaging Source)	1	500	500
Machine vision lens - low-mag	T3Z3510CS (Computar)	1	74	74
Objective lens	AC508-100-A-ML (Thorlabs)	1	160	160
Teleconverter lens (f = -100 mm)	ACN254-100-A (Thorlabs)	1	99	99
Teleconverter lens (f = 300 mm)	AC254-300-A-ML (Thorlabs)	1	114	114
Tube lens (f = 150 mm)	AC254-150-A-ML (Thorlabs)	1	114	114
Warm white LED flood	(Oznium)	1	16	16
**Lid handler**				
3D-printed tube holder socket pair	(Xometry)	2	33	66
Linear actuator	PQ12-30-12-S (Actuonix)	2	65	130
Solenoid valve	H2U29-00Y (Granzow)	2	217	434
Tube fitting	51025K196 (McMaster-Carr)	2	10	20
Vacuum tube - 10 ft.	52375K11 (McMaster-Carr)	1	9	9
**Autofocusing system**				
532 nm laser line generator	Quarton Laser Module VLM-532-47 LPT (Amazon)	1	77	77
Lens (f = 100 mm)	LA1509-A (Thorlabs)	1	36	36
**Plate tray**				
Aluminum layer	Reynolds Wrap (Amazon)	1	11	11
Laser-cut acrylic sheet	(Ponoko)	16	21	336
**Plate tracking system**				
Achromatic doublet lens	AC254-075-A-ML (Thorlabs)	1	114	114

Camera lens (f = 8 mm)	Computar	1	150	150
Machine vision camera	DMK33GP031 or DMK33GR0521(Imaging Source)	1	500	500
Warm white LED flood	(Oznium)	2	16	32
**Electronics control**				
DPDT relay - capacitive/heating circuit breaker	1885-1706-ND (Digi-Key)	1	22	22
DPDT relay - linear act. @ lid handler	mxuteuk HH52P DC 12V Coil 8 Pin 5A (Amazon)	2	12	24
Driver - 470 & 565 nm LED	LEDD1B (Thorlabs)	2	348	696
Driver - linear act. @ picking arm	TIC-T825 (Actuonix)	1	70	70
Driver - servos @ picking arm	RO-902-0132-000 (Trossen Robotics)	1	32	32
Relay driver	LJTick-RelayDriver (LabJack)	4	18	72
Solid state relay - heating circuit	6325AXXMDS-DC3 (Schneider)	1	128	128
SPST relay - autofocus laser; bright field illumination; barcode illumination; vacuum solenoid valve	2903361 (TTI)	5	10	50
USB DAQ device	U3-HV (LabJack)	1	155	155
		**Est. Total**		**$12,975**

Table S4. Fluorescence imaging and processing parameters. $\boldsymbol{T_e}$, $\boldsymbol{h_i}$, and $\boldsymbol{h_s}$ denote the exposure time, intensity threshold, and size threshold.

**Experiment**	**Purpose**	**Fluorescent transgene of interest**	**Imaging and processing parameters**		
			$\boldsymbol{T_e}$ (ms)	$\boldsymbol{h_i}$ (a. u.)	$\boldsymbol{h_s}$ (pixels)
Genetic cross (Fig. 3)	Assay transgene expression	*vsIs28 [dop-1p::GFP]*	200	70	20
Genetic mapping (Fig. 4)	Test I/III-linkage	*vsIs33 [dop-3p::RFP]*	100	80	20
		hT2 {qIs48 [myo-2::GFP]}	200	80	20
	Test II-linkage	*vsIs33 [dop-3p::RFP]*	100	80	50
		mIn1 {mIs14 [myo-2::GFP]}	200	60	50
	Test III/V-linkage	*vsIs33 [dop-3p::RFP]*	100	60	20
		eT1 {umnIs12 [myo-2::GFP]}	200	130	20
	Test IV/V-linkage	*vsIs33 [dop-3p::RFP]*	100	80	20
		nT1 {qIs51 [myo-2::GFP]}	200	80	20
	Test X-linkage	*vsIs33 [dop-3p::RFP]*	100	80	20
		qnEx615 [myo-2p::GFP]	200	80	20
Genomic integration (Fig. 5)	Assay transgene expression	*qhEx265 [unc-47p::GFP]*	200	70	1

Table S5. Pick motion trajectory parameters. $\boldsymbol{v_z}$ and $\boldsymbol{D_z}$ denote the velocity and the displacement of the component in $\boldsymbol{z}$ direction; $\boldsymbol{\alpha}$ and $\boldsymbol{\delta}$ denote the angular velocity and the angular displacement of the servo motor.

**Phase**	**Linear actuator**		**Servo 1**		**Servo 2**		**Servo 3**		**Main gantry**	
	$\boldsymbol{v_z}$ (mm/s)	$\boldsymbol{D_z}$ (mm)	$\boldsymbol{\alpha}$ (deg/s)	$\boldsymbol{\delta}$ (deg)	$\boldsymbol{\alpha}$ (deg/s)	$\boldsymbol{\delta}$ (deg)	$\boldsymbol{\alpha}$ (deg/s)	$\boldsymbol{\delta}$ (deg)	$\boldsymbol{v_z}$ (mm/s)	$\boldsymbol{D_z}$ (mm)
**Picking up**										
i	-20	-2.5	0	0	0	0	0	0	0	0
ii	0	0	0	0	274.8	1.23	0	0	0	0
iii	0	0	274.8	0.44	0	0	274.8	0.44	0	0
**Putting down**										
i	-20	-2.5	0	0	0	0	0	0	0	0
i-ii waiting (s)	1									
ii	0	0	0	0	-274.8	-1.58	0	0	0	0
ii-iii waiting (s)	2									
iii	0	0	0	0	0	0	0	0	-125	-0.4
iv	0	0	274.8	39.11	0	0	0	0	0	0

Table S6. Descriptions of the source scripts in WormPickerLib.

Category	Name	Description
High-level	CrossWorms	Pick hermaphrodites and males matching the specified phenotypes from two source plates to one destination plate.
	ScreenPlates	Screen multiple plates for assaying population phenotypes.
	SingleWorms	Pick worms matching the specified phenotypes to individual plates. A single worm per plate.
Mid-level	PickNWorms	Pick multiple worms matching the specific phenotypes from one plate to another.
	ScreenOnePlate	Screen a single plate for assaying population phenotypes.
Low-level	AutoFocus	Focus the image.
	CalibOx	Calibrate how many pixel-translations in the image corresponding to one unit movement in the gantry.
	CalibPick	Calibrate the pick position in the low-magnification FOV.
	CalibTray	Calibrate the plate positions over the plate tray platform.
	CenterWorm	Track worms and move individual animals to the high-magnification FOV.
	HeatPick	Sterilize the pick.
	MoveToPlate	Move the gantry to right above a plate.
	Phenotype	Phenotype any worms found in the high-magnification FOV.
	ReadBarCode	Read the barcode identifier for a plate, write/read the plate information to/from the database.
	SpeedPick	Perform a single pick-up/put-down action.

Table S7. Strains used in this study.

Name	Source or reference	Identifiers	Additional information
OP50	CGC	Fang-Yen Lab Strain Collection: OP50 RRID:WB-STRAIN:WBStrain00041971	OP50
CB1091	CGC	Fang-Yen Lab Strain Collection: CB1091	unc-13(e1091) I.
CB61	CGC	Fang-Yen Lab Strain Collection: CB61	dpy-5(e61) I.
CGC34	Gift of D. Raizen	Fang-Yen Lab Strain Collection: CGC34	eT1 [umnIs12] III; eT1 V.
JK2810	Gift of D. Raizen	Fang-Yen Lab Strain Collection: JK2810	mcm-4(e1466) dpy-5(e61)/hT2 I; dpy-18(e364) III/hT2 [bli-4(e937) let-?(q782) qIs48] (I;III).
JK2958	Gift of D. Raizen	Fang-Yen Lab Strain Collection: JK2958	nT1 [qIs51] (IV;V)/dpy-11(e224) unc-42(e270) V.
LX811	CGC	Fang-Yen Lab Strain Collection: LX811	vsIs33 V; lin-15B&lin-15A(n765) X.
LX831	CGC	Fang-Yen Lab Strain Collection: LX831	vsIs33 V; lin-15B&lin-15A(n765) X; vsIs28.
N2	CGC	Fang-Yen Lab Strain Collection: N2	wild-type
NC1750	Fang-Yen Lab	Fang-Yen Lab Strain Collection: NC1750	glr-1p::DsRed2 / opt-3::GFP + rol-6(su1006)
NQ1155	Gift of D. Raizen	Fang-Yen Lab Strain Collection: NQ1155	qnEx615 [myo-2p::GFP]
UP2436	Gift of M. V. Sundaram	Sundaram Lab Strain Collection: UP2436	lpr-1(cs207) I.
VC170	Gift of D. Raizen	Fang-Yen Lab Strain Collection: VC170	cki-1(gk132)/mIn1 [dpy-10(e128) mIs14] II.

VM6365	Fang-Yen Lab	Fang-Yen Lab Strain Collection: VM6365	lin-15 (n765ts); akEx387[lin-15(+), dat-1::GFP, dat-1::ICE]
YX256	Fang-Yen Lab	Fang-Yen Lab Strain Collection: YX256	syEx723 [hs::LIN-3C; myo-2::GFP; pha-1(+)]
YX293	Fang-Yen Lab	Fang-Yen Lab Strain Collection: YX293	dop-3(vs106); qhEx265 [acr-2p::DOP-3(+) + unc-47p::GFP]
YX300	Crossed LX831 with CB61	Fang-Yen Lab Strain Collection: YX300	vsIs28 [dop-1p::GFP]; dpy-5 (e61) I.
YX301	UV integration of YX293	Fang-Yen Lab Strain Collection: YX301	dop-3(vs106); qhIs12 [acr-2p::DOP-3 + unc-47p::GFP]
YX302	UV integration of YX293	Fang-Yen Lab Strain Collection: YX302	dop-3(vs106); qhIs13 [acr-2p::DOP-3 + unc-47p::GFP]

Movie S1 (separate file). Automated multimodal imaging of *C. elegans.* Transgenic (JK2958) *C. elegans* in bright field images followed by an image from the GFP channel. Transgenic (LX811) *C. elegans* in bright field images followed by an image from the RFP channel. FOV: 1.88 mm x 1.57 mm.

Movie S2 (separate file). Lid manipulation. Lids of 6-cm diameter agar plates on the platform are removed and replaced using two motorized vacuum actuators.

Movie S3 (separate file). Pick sterilization. The platinum wire loop pick is sterilized by resistive heating.

Movie S4 (separate file). Automated *C. elegans* picking from the WormPicker's camera view. Overlay of the high-magnification and low-magnification video streams. A worm is automatically picked up by the wire loop pick and transferred to a different plate.

Movie S5 (separate file). Automated *C. elegans* transfer between 6-cm diameter agar substrates. An electric current sterilizes the pick; worms are tracked and phenotyped; the robotic arm picks up a worm from a plate and transfers it to a second plate.

Data Set S1 (separate file). Source data. The file contains the source data for the plots presented in this paper.

Design File S1 (separate file). WormPicker mechanical design file. The file contains a CAD design (F3D format) for the WormPicker hardware system, including a plate tray platform, X, Y, Z linear carriage assemblies, an optical imaging system, two lid handlers, a robotic picking arm, an autofocusing system, an illuminator (under the platform), and a plate tracking system (under the platform).

References for the supplementary material

1. K. He, G. Gkioxari, P. Dollár, R. Girshick (2017) Mask r-cnn. in *Proceedings of the IEEE international conference on computer vision*, pp 2961-2969.
2. A. M. Leifer, C. Fang-Yen, M. Gershow, M. J. Alkema, A. D. Samuel, Optogenetic manipulation of neural activity in freely moving Caenorhabditis elegans. *Nat. Methods* **8**, 147-152 (2011).
3. L. Byerly, R. Cassada, R. Russell, The life cycle of the nematode Caenorhabditis elegans: I. Wild-type growth and reproduction. *Dev. Biol.* **51**, 23-33 (1976).
4. K. S. McKim, K. Peters, A. M. Rose, Two types of sites required for meiotic chromosome pairing in Caenorhabditis elegans. *Genetics* **134**, 749-768 (1993).
5. M. Edgley, D. Riddle, LG II balancer chromosomes in Caenorhabditis elegans: mT1 (II; III) and the mIn1 set of dominantly and recessively marked inversions. *Mol. Genet. Genomics* **266**, 385-395 (2001).
6. R. E. Rosenbluth, D. L. Baillie, The genetic analysis of a reciprocal translocation, eT1 (III; V), in Caenorhabditis elegans. *Genetics* **99**, 415-428 (1981).
7. R. E. Rosenbluth, C. Cuddeford, D. L. Baillie, Mutagenesis in Caenorhabditis elegans. II. A spectrum of mutational events induced with 1500 r of γ-radiation. *Genetics* **109**, 493-511 (1985).
8. E. L. Ferguson, H. R. Horvitz, Identification and characterization of 22 genes that affect the vulval cell lineages of the nematode Caenorhabditis elegans. *Genetics* **110**, 17-72 (1985).
9. D. V. Clark, T. M. Rogalski, L. M. Donati, D. L. Baillie, The unc-22 (IV) region of Caenorhabditis elegans: genetic analysis of lethal mutations. *Genetics* **119**, 345-353 (1988).
10. T. M. Rogalski, D. L. Riddle, A Caenorhabditis elegans RNA polymerase II gene, ama-1 IV, and nearby essential genes. *Genetics* **118**, 61-74 (1988).
